# Effects of liquid cultivation on gene expression and phenotype of *C. elegans*

**DOI:** 10.1186/s12864-018-4948-7

**Published:** 2018-07-31

**Authors:** İrem Çelen, Jung H. Doh, Chandran R. Sabanayagam

**Affiliations:** 1 Center for Bioinformatics and Computational Biology, University of Delaware, Newark, DE 19711 USA; 2 Delaware Biotechnology Institute, University of Delaware, 15 Innovation Way, Newark, DE 19711 USA

**Keywords:** Dormant genes, ncRNA, Adaptation, Reaction norm

## Abstract

**Background:**

Liquid cultures have been commonly used in space, toxicology, and pharmacology studies of *Caenorhabditis elegans*. However, the knowledge about transcriptomic alterations caused by liquid cultivation remains limited. Moreover, the impact of different genotypes in rapid adaptive responses to environmental changes (e.g., liquid cultivation) is often overlooked. Here, we report the transcriptomic and phenotypic responses of laboratory N2 and the wild-isolate AB1 strains after culturing P_0_ worms on agar plates, F_1_ in liquid cultures, and F_2_ back on agar plates.

**Results:**

Significant variations were found in the gene expressions between the N2 and AB1 strains in response to liquid cultivation. The results demonstrated that 8–34% of the environmental change-induced transcriptional responses are transmitted to the subsequent generation. By categorizing the gene expressions for genotype, environment, and genotype-environment interactions, we identified that the genotype has a substantial impact on the adaptive responses. Functional analysis of the transcriptome showed correlation with phenotypical changes. For example, the N2 strain exhibited alterations in both phenotype and gene expressions for germline and cuticle in axenic liquid cultivation. We found transcript evidence to approximately 21% of the computationally predicted genes in *C. elegans* by exposing the worms to environmental changes.

**Conclusions:**

The presented study reveals substantial differences between N2 and AB1 strains for transcriptomic and phenotypical responses to rapid environmental changes. Our data can provide standard controls for future studies for the liquid cultivation of *C. elegans* and enable the discovery of condition-specific genes.

**Electronic supplementary material:**

The online version of this article (10.1186/s12864-018-4948-7) contains supplementary material, which is available to authorized users.

## Background

All living systems possess the fundamental property to regulate physical or genetic states in response to environmental stimuli. *Caenorhabditis elegans* offers one of the unique models to study the effects of environmental changes as it can reproduce in large numbers and has a natural ability to live in both solid and liquid conditions. This eukaryotic organism provides the opportunity to study genetic mechanisms in the whole animal rather than a cell culture. In addition, it can consume a bacterial or an axenic food source thereby enabling the identification of diet-related biological processes. Placing the worms from bacteria seeded agar plates (OP50 NGM) to liquid axenic media poses a drastic change for the animals. For example, the animals display locomotive differences in the liquid by swimming via a rapid C-shape thrashing motion as opposed to crawling via a sinusoidal configuration on solid [1]. Behavior and physiological differences arise when the worms are grown in liquid axenic media (such as CeMM; *C. elegans* Maintenance Medium or CeHR; *C. elegans* Habitation and Reproduction) [2, 3]. In axenic media, the worms exhibit phenotypical alterations such as delayed development, reduced fecundity, increased longevity, and altered body morphology [4–6]. Environment changes can trigger different gene expression profiles, and these profiles can be transmitted to the subsequent generations [7–9]. Changing the environment of the animals from agar to axenic media, therefore, can cause transcriptome-level alterations which may have a sustained effect on the progenies. However, the knowledge about transcriptomic responses to axenic media is limited. For example, in their microarray analysis, Szewczyk et al. could manage to obtain reliable data for only 1202 cDNAs on all three arrays consistently for CeMM-grown worms [5]. The common usage of axenic media in space, toxicology, and pharmacology studies highlights the necessity of a complete transcriptomic analysis, preferably with RNA-sequencing (RNA-seq) [6, 10].

In its natural habitat, *C. elegans* occupies microbiota-rich decaying plant material and experiences a continuous environment shift between liquid and solid conditions along with the variations in its diet [11]. The wild-type laboratory strain of *C. elegans* (N2) has been cultured in both liquid and solid conditions [12], but bacteria-seeded agar plates are used more commonly [13]. It has been reported that the domestication of the N2 strain has created a selective pressure on the animals [14]. Some of the critical questions that await answers are whether: 1.) the adaptive responses depend on the genotype or the environment change; 2.) the environment change-induced gene expression profiles are transmitted to the successive generation in equal levels for different genotypes; 3.) the environment change-induced gene expression profiles are transmitted to the next generation equally for different environmental conditions.

To study the maternal effects on transcriptomic responses of *C. elegans* from solid to liquid environments, we maintained the P_0_ worms on bacteria-seeded agar plates and F_1_ in liquid cultures. By culturing F_2_ generation back on agar, we identified the maintained maternal transcriptional responses. The N2 strain and a wild isolate AB1 strain were used to examine if a different genotype attributes to the transcriptional dynamics. It is important to note that even though the N2 and the AB1 strains are relatively close to each other in the phylogenetic tree [15], AB1 is not the ancestral strain of N2. Therefore, the observed differences in norm of reaction can stem from the natural genetic differences between N2 and AB1 as much as the impact of laboratory domestication on adaptive responses. We found substantial variances in gene expression profiles resulting from different genotypes, environments, and genotype-environment interactions. Our results from RNA-seq data analysis and microscopy showed consistency for the predicted and observed phenotype. Furthermore, we demonstrated that a large number of genes are expressed only in specific growth conditions, and many of these genes previously lacked experimental expressed sequence tag (EST) or cDNA evidence.

## Results

To induce an adaptive response and observe its sustained effects on the following generation, the P_0_ generation *C. elegans* was grown on agar, the F_1_ generation in liquid, and the F_2_ generation back on agar (Additional file 1: Figure S1a). We carried out three sets of experiments with different food sources and animal strains. For the first experiment, the F_1_ generation N2 strain was grown in axenic CeHR Medium. To examine the effect of dietary changes in the liquid cultures, we cultured the F_1_ generation N2 strain in bacterial S-Medium for the second experiment. Finally, the transcriptomic response differences were investigated by growing F_1_ generation AB1 strain in CeHR Medium. For each generation, RNA-seq was performed on young adult animals with three replicates to determine the transcriptional responses (Additional file 1: Table S1).

### Distinct gene groups are expressed exclusively in specific environmental conditions

**Fig. 1:**
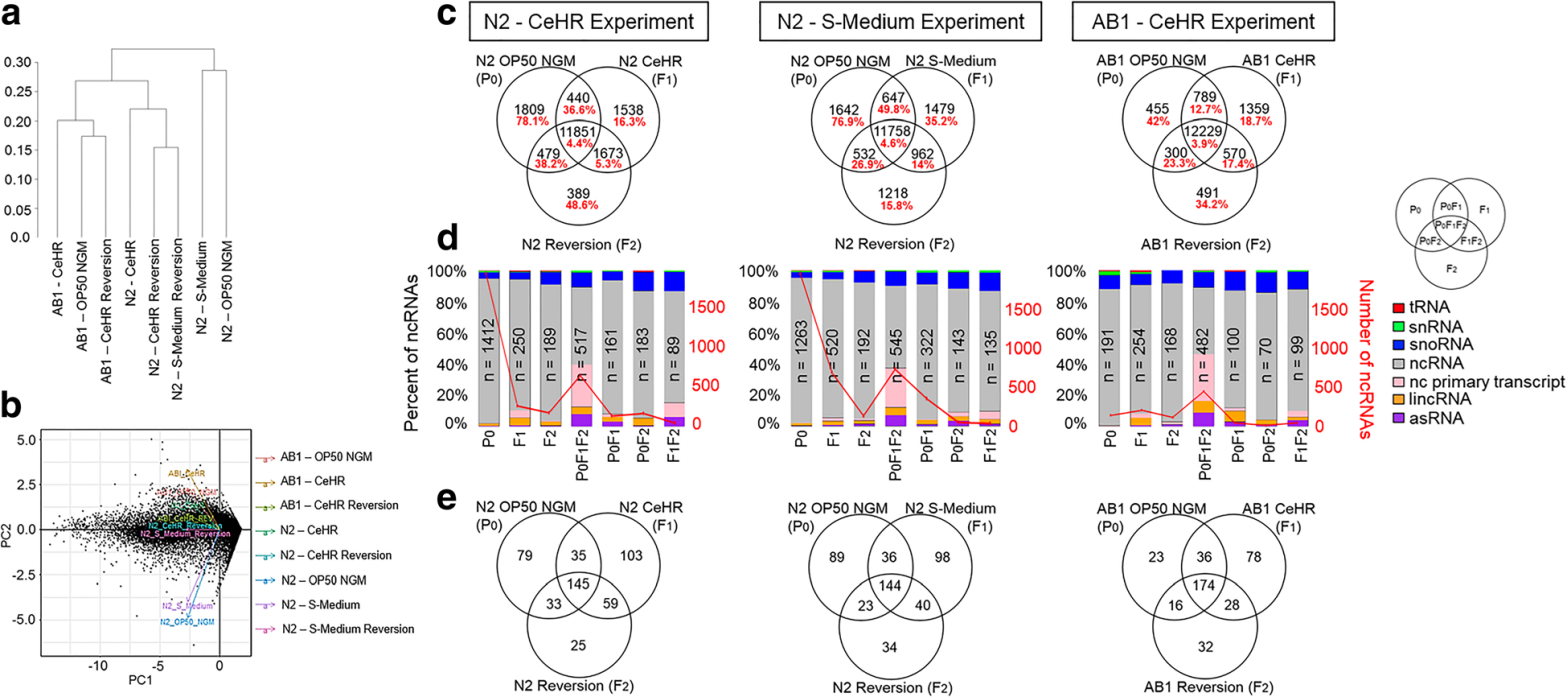
Different strains and environmental conditions present variations in transcriptional responses. **a** Dendrogram created based on the gene expression (FPKM) values for the strains and the environmental conditions. **b** Principal component analysis plot of gene expression data. **c** Categorization of the expressed genes in the three experiments. Red labels represent the percentages of ncRNAs. **d** The barplots demonstrate ncRNA profiles in the three experiments. **e** Categorization of the previously unconfirmed genes that show expression in our experiments

The transcriptome-based clustering of the experiments in the dendrogram of Jensen-Shannon distances demonstrated that the genotypes are separated from each other (Fig. 1a). Exposure to different environmental conditions causes the N2 transcriptome to be separated even further than the AB1 transcriptome as illustrated by the dendrogram and principal component analysis (Fig. 1a, b, Additional file 1: Figure S1b). These results suggest that the genotype plays a key role in adaptive response, and the different strains of the animals can show highly distinct transcriptome profiles under environmental changes. We categorized the genes by using Venn diagrams to identify if their expressions are condition specific (Fig. 1c). We considered the genes expressed if their Fragments Per Kilobase of transcript per Million mapped reads (FPKM) values are greater than one as this is a commonly used criterion for expression [16–19]. The majority of the expressed genes are conserved among the P_0_, F_1_, and F_2_ generations. Nevertheless, hundreds of genes are expressed only under particular conditions. The number of the uniquely expressed genes showed a significant difference between CeHR grown AB1 and N2 strains, but not within the N2 strain experiments (Chi-squared test with Yates correction; *P-value* = 0.01 and *P-value* = 0.9, respectively). In addition, the N2 strain showed an overall decrease in the transcription levels when exposed to the changes in growth conditions (Additional file 1: Figure S2). AB1 strain, however, demonstrated an increase in the total FPKM but a decrease in the average FPKM under CeHR (Additional file 1: Figure S2). Interestingly, the number of gene expression patterns transmitted to the next generation after exposure to an environment change (F_1_F_2_) varies among the experiments (Chi-squared test with Yates correction; *P-value* < 0.001). Together, these results seem to suggest that the transmission of the gene expression is not exclusively dependent on the genotype and the environment separately—but dependent on their interaction, whereas the condition-specific expression of genes is affected by the genotype.

Further analyses revealed that non-coding RNA (ncRNA) molecules are enriched (16–78%) in our pool of condition-specific transcripts indicating their function in rapid adaptive responses (Fig. 1c). To note, only the polyadenylated ncRNA molecules were included in the analysis, and piRNA and miRNA molecules were excluded from the analysis due to their short lengths. The ncRNA molecules were predominantly observed in the P_0_ generation of N2 strain compared to the P_0_ AB1 strain. Moreover, fewer ncRNA molecules were expressed when the animals are exposed to an environment change (Fig. 1c*,* d, and Additional file 1: Figure S3). Our results indicate that ncRNA expression is mainly silenced when the worms are exposed to the tested environmental changes. The fact that a laboratory condition presents an environment change for the wild-isolate may explain why P_0_ animals of AB1 showed less ncRNA expression compared to the N2 strain. One particular exception to this pattern is the ncRNA molecules that are commonly expressed in all three generations. We identified that the majority of these ncRNAs (59–66%, *n* = 322) are conserved among the three experiments (Additional file 2: Dataset S1). This conservation implies that this group of ncRNAs plays important regulatory functions in the cell and their expression is required regardless of the environmental changes. Overrepresented phenotype for these ncRNAs is dendrite development variant (FDR adj. *p-value* < 0.01, six-fold enrichment) suggesting a potential role for them in generation of neuronal extensions.

We reasoned that if a particular group of genes is only expressed under specific conditions, we might detect transcript evidence to computationally predicted but experimentally unconfirmed genes, especially considering that CeHR is not a common medium used in *C. elegans* research. Notably, we were able to identify transcript evidence to 21% of the list of unconfirmed genes from WormBase [20] by simply growing the animals in different environmental conditions (Fig. 1e). Unexpectedly, a substantial amount of the transcript evidence was found with animals raised in commonly used OP50 NGM. We compared our list of expressed unconfirmed genes in OP50 NGM-grown N2 to those from a previous study [21] and found that 54% of the unconfirmed genes from our list (*n* = 292) have also shown expression in the study by Dallaire et al. but were not reported as transcript evidence in WormBase. The remaining 46% of the genes must have been induced due to the subtle differences introduced in laboratory environment (e.g., light and environment). Similar to the confirmed genes, gene expression transmission to the successive generation after environmental change demonstrated a difference between and within the strains (Chi-squared test with Yates correction; *P-value* < 0.001, *P-value =* 0.02, respectively). Given that it can robustly provide transcript evidence to unconfirmed genes, our approach and data can be a rich resource for discovery of novel genes.

### Environment, genotype, and environment-genotype specific expression of genes

**Fig. 2:**
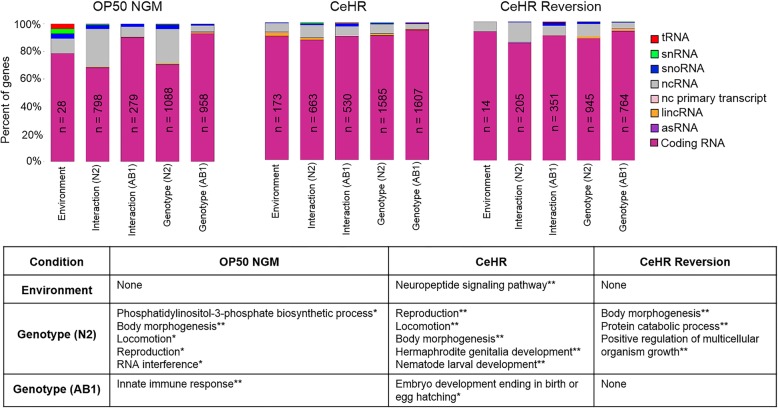
Shown are the number of gene expressions exclusive to environment, genotype, or genotype-environment interactions. The number of the expressed ncRNAs are decreased under stress conditions. The table depicts the enriched gene ontology (GO) terms assigned to environment or genotype specifically expressed genes. Genotype-environment interaction genes did not demonstrate enrichment for GO terms. “None” represents the groups with no GO enrichment. Benjamini Hochberg corrected *P-values* for the GO terms: * < 0.01; ** < 0.001

We grouped the genes based on their genotype, environment, or genotype-environment interaction specific expressions (Fig. 2 and Additional file 3: Dataset S2). For this analysis, a more stringent criterion was used to eliminate any potential noise from the data. That is, we only considered the genes with expression levels higher than *pmp-3* of FPKM = 21, and excluded genes with FPKM < 21 from the analysis. This housekeeping gene was selected as a reference because of its stable expression levels found both in our analysis and previous studies [22, 23]. Specifically for the ncRNA molecules, we reasoned that expression levels higher than that of a housekeeping gene should provide strong evidence for expression.

We defined the genes that are expressed in only one environmental condition for both the strains as “environment-specific”, and found that CeHR-specific gene expression displayed Gene Ontology (GO) enrichment for the neuropeptide signaling pathway. Given that this pathway functions in numerous behavioral activities such as reproduction, locomotion, mechanosensation and chemosensation, it is likely that the neuropeptide signaling pathway plays a crucial role in rapid adaptation to CeHR and is evolutionarily conserved between the strains [24].

The “genotype-specific” expression represents the genes found in only one of the strains under a single environmental condition. All the N2 specific genes showed GO enrichment for body morphogenesis in all the three generations while GO enrichment for reproduction and locomotion were observed in P_0_ and F_1_ generations (Fig. 2). Previously, we reported that the N2 worms produce less progeny compared to the AB1 animals in CeHR [6]. Our analysis showed that along with the reproduction genes, genes related to hermaphrodite genitalia development were expressed exclusively in the N2 strain in CeHR. This finding may suggest vulva aberrations in the reproductive system. In addition, the exclusive expression of the genes functioning in positive regulation of multicellular organism growth may help the animals readapt to the OP50 NGM condition in the F_2_ generation of the N2 animals.

The genes expressed solely under a particular environment and strain were categorized as “genotype-environment interaction” genes (Fig. 2). This group of genes did not demonstrate an enrichment of GO terms. The proportion of ncRNA molecules in our group of genes showed correlation with our hypothesis that the majority of the ncRNAs are silenced under the tested environmental changes. For example, the N2 worms showed relatively higher proportions of ncRNAs for interaction and genotype-related genes on OP50 NGM, but these proportions were much lower in more stressful CeHR and CeHR reversion (Fig. 2).

### N2 strain presents higher differential expression under environment changes

**Fig. 3:**
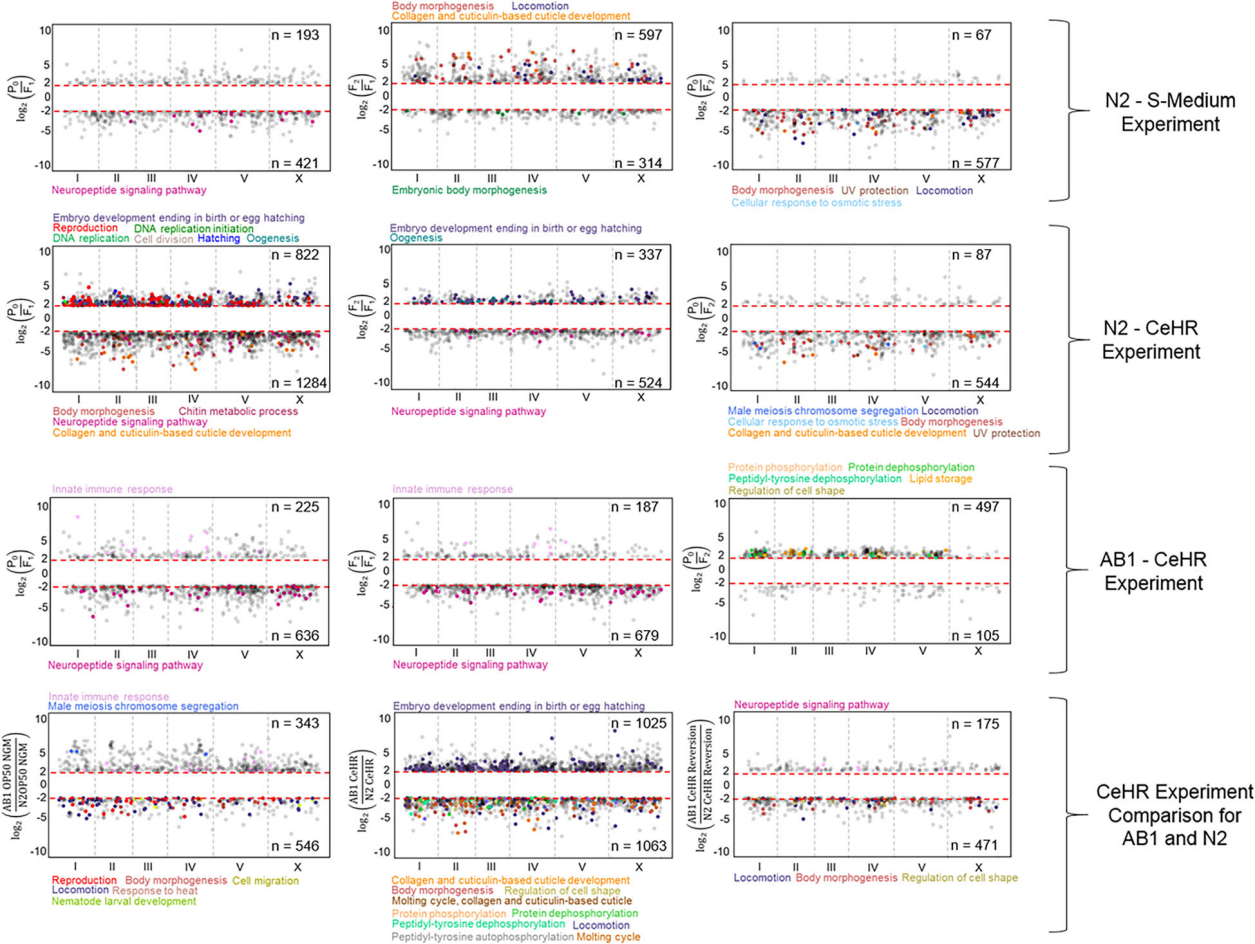
The differential expression of the genes (FPKM > 1, FDR adj. *P-*value < 0.05, and log2(fold change of the FPKM values) ≥ 2) between the environment conditions and the strains. Red dashed lines represent the two-fold cutoff for differential gene expression. The corresponding genes for the enriched GO terms were represented with the colors. Benjamini Hochberg corrected and FDR adj. *P*-values < 0.05 for all the enriched GO terms. The GO terms were determined separately for downregulated and upregulated genes

To investigate the differences in transcriptional responses between the strains and among the environmental conditions, we identified the differentially expressed genes (DEGs). We considered genes as differentially expressed if their FPKM is greater than one and their expression is altered over two-folds among the conditions with FDR-adjusted *p-values* ≤ 0.05. The estimated fold change responses of 20 randomly selected transcripts (ten for OP50 NGM against CeHR and ten for CeHR against CeHR reversion) were confirmed by quantitative RT-PCR (Additional file 1: Figure S4a, b and Additional file 1: Table S2 and S3). We found the highest number of DEGs between OP50 NGM and CeHR conditions for the N2 strain (Fig. 3). This indicates that the change in diet and physical environment triggers more transcriptional responses for adaptation of the N2 strain. The number of DEGs were much lower for the F_2_ generation of the same experiment, suggesting that the majority of the gene expressions started to resemble the P_0_ animals. This finding was expected as most of the gene expression patterns return to the steady-state levels after a short period [25]. The change in only the physical condition introduced by the S-Medium apparently did not cause as drastic transcriptional responses as the CeHR since the number of the DEGs were lower (38 and 45% lower number of upregulated and downregulated genes, respectively). However, S-Medium reversion conditions demonstrated higher DEGs compared to OP50 NGM. We note that we are able to maintain the worms in S-Medium for only one generation, potentially because of the toxins released by the bacteria in the liquid environment [26, 27]. Therefore, additional genetic mechanisms may be required for re-adaptation of the animals from S-Medium. Transcriptional variations were more modest across the generations for AB1 animals than the N2 strain.

Substantial discrepancies have been reported for the *C. elegans* gene expressions among axenic media studies [28]. We wished to compare our results to those from CeMM-grown worms as CeMM has a very similar base medium to CeHR. For the 21 upregulated and 26 downregulated genes reported in a previous study [5], we could only identify one common downregulated gene in N2 and AB1 and four and seven common upregulated genes in N2 and AB1, respectively (Additional file 1: Table S4). It is unclear why the DEGs among the axenic media studies mostly disagree, but some potential reasons can be the difference in the technology used and the modifications made in the media. For instance, microarray technology has been reported to introduce more background noise and cross-hybridization and detect a lesser number of DEGs compared to RNA-seq [29]. A more unified study conducted with RNA-seq may help resolve the discrepancy issues among the axenic media experiments.

In our previous study, we have shown that CeHR-grown N2 and AB1 *C. elegans* display different phenotypical traits on their body morphology [6]. That is, adult animals of both strains demonstrated physical differences in CeHR compared to the OP50 NGM-grown ones, but the CeHR-grown AB1 strain was even longer and thinner than the CeHR-grown N2 strain in average (N2 length is 825.2 ± 74.3 μm and width is 45.9 ± 10.1 μm, and AB1 length is 876.8 ± 67.0 μm and width is 38.3 ± 4.7 μm). In accordance with this finding, the GO enrichment analysis results demonstrated enrichment for body morphogenesis for upregulated genes in CeHR compared to OP50 NGM in the N2 strain, but not the AB1 strain (Fig. 3). Moreover, genes upregulated under the CeHR condition in the AB1 strain compared to the N2 strain were overrepresented for body morphogenesis. The fact that this GO term is not enriched for AB1 may imply that the phenotype is caused by a separate transcriptional mechanism. To test this, we acquired the genes associated with “long” and “thin” phenotypes from WormBase [20] and compared these genes to the DEGs in our list. These phenotypes are generally found by RNAi knockdown of genes. We found that six and nineteen differentially expressed genes functioning in long or thin phenotypes in N2 and AB1, respectively, but only one gene, *his-67*, was common between the strains (Additional file 1: Table S5). Thus, it is possible that the different gene expression profiles contribute to the body morphology variations between the strains.

Neuropeptide signaling pathway genes were enriched in response to environmental changes. The pathway genes were upregulated in the liquid environments compared to agar in N2 and AB1. The differential expression of this pathway genes in liquid conditions did not maintain their expression patterns in the next generation. This finding supports our hypothesis that neuropeptide signaling pathway can be a crucial mechanism in rapid adaptive responses and it is conserved in the different strains of *C. elegans*.

Given the component differences in OP50 NGM, CeHR, and S-Medium, it is expected to observe variations in the expressions of metabolic process genes among these conditions. We performed pathway enrichment analysis on DEGs to determine whether dietary sensor pathways, such as insulin/insulin-like growth factor (IGF) and target of rapamycin (TOR), are affected in the tested liquid cultivations. We have not observed enrichment for the renowned dietary sensor pathways but found other metabolic pathways to be affected. For instance, in CeHR, metabolic process in N2 and lysosome and fatty acid metabolism in AB1 demonstrated overrepresentation (Table 1). CeMM-grown animals have been reported not to show pathologies related to starvation [5]. Similarly, we have not detected differential expression for dietary restriction linked insulin-like signaling, *pha-4*/FoxA, *skn-1*/Nrf, or *nhr-49*/Hnf4 [30, 31]. Together, these results seem to suggest that CeHR does not trigger a starvation response in the worms.

**Table 1:**
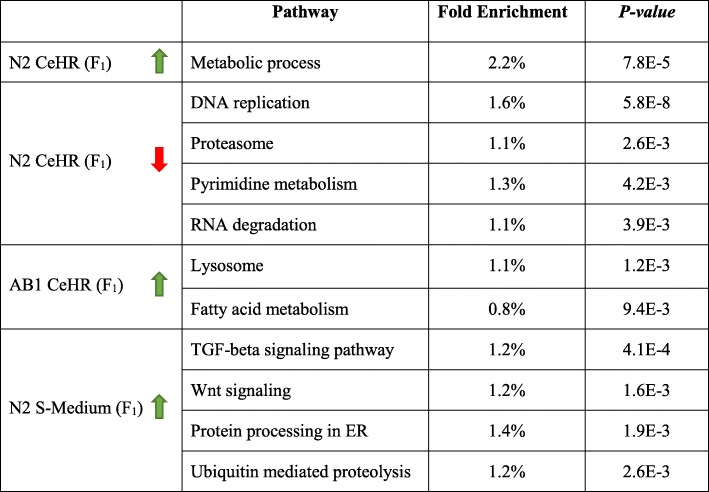
Enriched pathways in F1 generations in response to the liquid cultivations**.** The green and red arrows show upregulation and downregulation compared to the expression in the corresponding P_0_ generations. The *P-values* are Benjamini-Hochberg corrected.

**Fig. 4:**
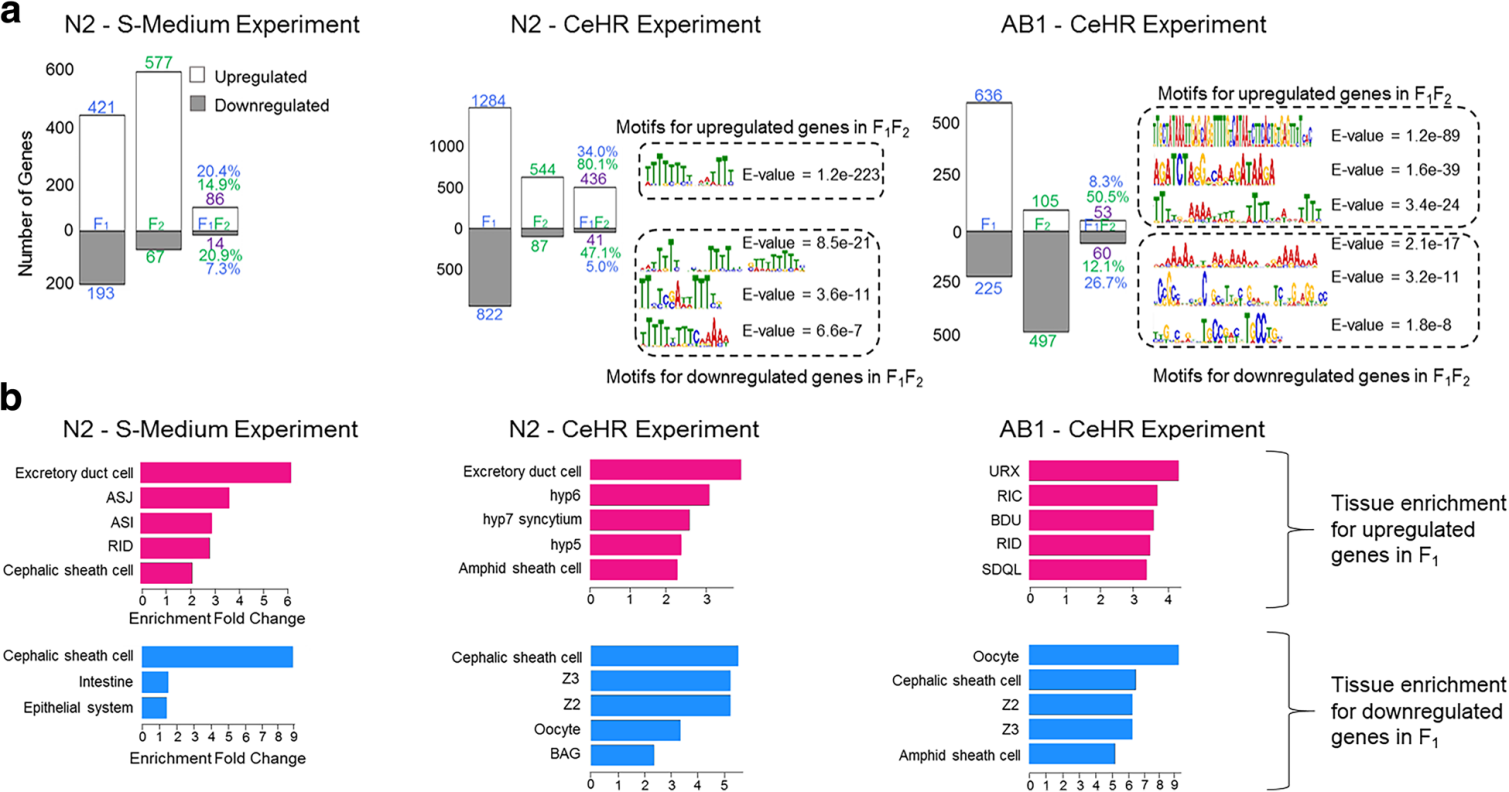
Different strains and the environment conditions contribute to differences in the maternal gene expression profiles. **a** The DEGs in the F_1_, F_2_, and both (F_1_F_2_) generations were identified in comparison to the gene expressions in the P_0_ animals. Between 5 to 34% of the DEGs in the F_1_ generations were transmitted to the F_2_ generations (see the third category – F_1_F_2_) and these transmissions are higher than expected by chance (hypergeometric test, *P-value* < 0.001 for all the upregulated and downregulated genes individually). The gene expressions transmitted to the next generation after exposure to CeHR showed distinct enrichments for sequence motifs in their promoter regions for N2 and AB1 strains. The number of DEGs genes in F_1_, F_2_, and F_1_F_2_ were represented with blue, green, and purple, respectively. **b** Top five tissue enrichment of the DEGs under exposure to environmental changes in F_1_ (FDR-adj*. P-value* < 0.05)

Particular environmental change-triggered DEGs in F_1_ maintained their upregulation or downregulation in the next generation. The transmission of the environment-induced differential gene expressions to the F_2_ generation from F_1_ was between 20 and 34% (Fig. 4a). To assess whether the transmitted differential gene expressions are orchestrated by the same transcriptional regulators (i.e., transcription factors), we first evaluated the promoter regions of these genes for motif enrichments as potential recognition sites. The S-Medium experiment did not present any motif enrichment while the CeHR did for multiple sequence motifs in both the strains. The enriched motifs from both the upregulated and downregulated genes demonstrated differences between the strains, indicating that distinct transcriptional regulators can potentially be in play. We further compared the motifs from the upregulated genes against known transcription factor recognition sites [32] to find if common transcription factors induce gene expressions in CeHR for the strains. One such motif from both the strains is a recognition site for transcription factor MDL-1 (Additional file 1: Figure S5a). This transcription factor has regulatory functions in the inhibition of germline growth and longevity [33]. Other motifs from upregulated genes did not exhibit enrichment for transcription factor recognition. To test the human biological processes related to the putative MDL-1 recognition motifs from both the strains, we performed GO analysis for the DNA motifs against human promoters [32]. Both the motifs were overrepresented with the sensory perception of smell GO term, presumably corresponding to chemosensation functioning in the detection of environmental changes in worms. However, other enriched terms were different for these two motifs. For instance, the motif from the N2 strain had an overrepresentation of DNA damage checkpoint and transcription initiation from RNA polymerase II promoter while the motif from the AB1 strain was enriched for G-protein coupled receptor protein signaling pathway and positive regulation of immune response (Additional file 1: Figure S5a).

We next sought to identify the transcriptional factor genes expressed at higher levels in the F_1_ and F_2_ generations. We considered the F_1_-induced transcription factor genes as the putative regulators of the environmental change responses and F_2_-induced ones as the putative regulators of re-adaptive responses. The comparison of our list of DEGs to the putative transcription factor genes [34] revealed that the majority of the differentially expressed transcription factors genes are strain and condition-specific (Additional file 1: Figure S5b). None of the CeHR reversion-induced transcription factor genes were common between the strains indicating different transcriptional re-adaptive responses to agar plates for the N2 and AB1 strains. We identified only five shared CeHR-enriched transcription factor genes between the strains, and six shared genes between the CeHR and S-Medium conditions in N2 (hypergeometric test; *p-value* < 0.001). Neuron and muscle cell enriched R06C1.6 was commonly induced in CeHR for both the strains and in S-Medium for N2, suggesting a role for R06C1.6 in swimming behavior.

To explore whether the DEGs among the F_1_ generations show similar spatial expressions, we performed tissue enrichment analysis (Fig. 4b). We discarded any enrichments on embryonic cells as we conducted all our experiments on the L4 stage worms. The downregulated genes in CeHR demonstrated enrichment for reproductive system-related tissues such as the oocyte, Z2, and Z3 for both the strains. This possibly reflects the changes in the reproduction of the worms in CeHR. However, upregulated genes in CeHR exhibited overrepresentation on different tissues between the strains: DEGs in N2 were mainly enriched for cylindrical hypodermal syncytium in head; DEGs in AB1 were enriched for neuronal tissues. The genes upregulated in response to S-Medium showed overrepresentation for neuronal tissues, but to different ones than the CeHR-exposed AB1 strain genes. Notably, downregulated genes in CeHR for both the strains and in S-Medium for N2 are enriched in cephalic sheath cell (Fig. 4b). Altogether, these findings suggest that laboratory and wild-isolate strain worms show significant variations in the transcriptomic responses to the environmental alteration.

### Functional profiles of the differentially expressed genes show correlation with the observed phenotype

**Fig. 5:**
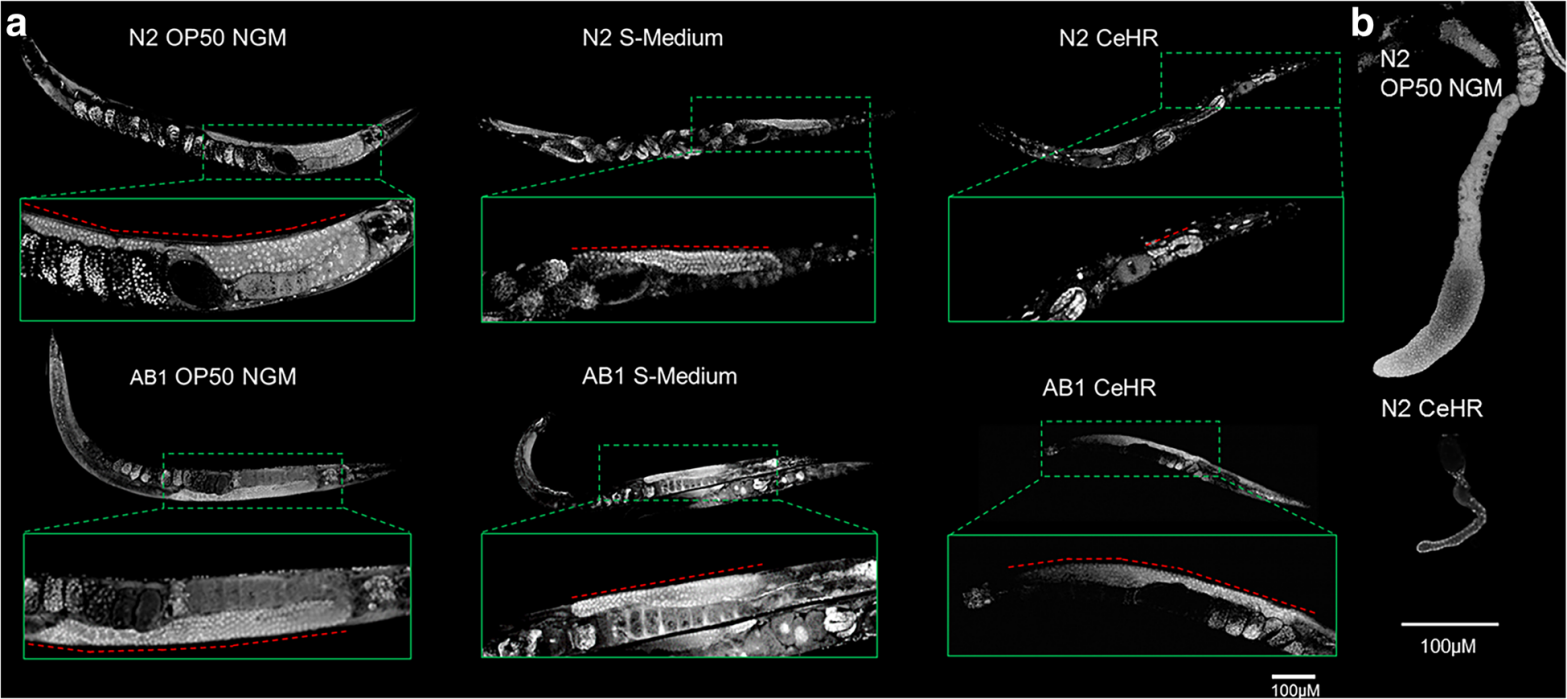
N2 but not AB1 *C. elegans* exhibit germline abnormalities in CeHR. **a** N2 and AB1 germlines exhibit normal germ cell morphologies and numbers in OP50 NGM (aberrant germlines were observed in 21 and 12.5% of the worms, respectively). Germlines of S-Medium grown animals are similar to OP50 NGM grown animals. However, embryos accumulate in 36% of the S-Medium grown adults. As expected, N2 adults grown in CeHR exhibit aberrant germlines with drastically reduced germ cell numbers, which results in an overall smaller germline (86% of the worms). N2 adults in CeHR have a significantly fewer number of oocytes and a high number of internally hatched larvae. These abnormalities are not observed in CeHR-grown AB1 adults (aberrant germlines were observed in 33% of the worms). **b** Dissected out gonadal arms of N2 *C. elegans* grown on OP50 NGM and CeHR. Germline size and cellular morphology are severely deformed in animals grown in CeHR

We wondered if the functional data analysis results indeed point to phenotypical alterations in the animals. To test this, we employed microscopy techniques to detect the potential phenotypes. In the N2 strain, the genes functioning in reproduction and oogenesis have been downregulated in CeHR compared to agar and reversion. In AB1, the DEGs among the conditions did not show the same GO enrichment. This pattern is an indicator of phenotypical changes in the reproductive system that can be observed in CeHR but absent in reversion for N2. To test our prediction, we examined the germlines of N2 and AB1 adults in the different growth conditions via confocal microscopy (Fig. 5a, b, Additional file 1: Figure S8). CeHR grown N2 but not AB1 adults displayed aberrant germlines. Additionally, fewer oocytes were observed in CeHR-grown N2 strain compared to OP50 NGM-grown N2 strain. These findings suggest that the reduced fecundity in CeHR raised N2 animals may have resulted from the inability of animals to make gametes. In agreement with our hypothesis, the worms placed back to agar plates did not exhibit the similar phenotypical abnormalities with CeHR grown animals (Additional file 1: Figure S6).

**Fig. 6:**
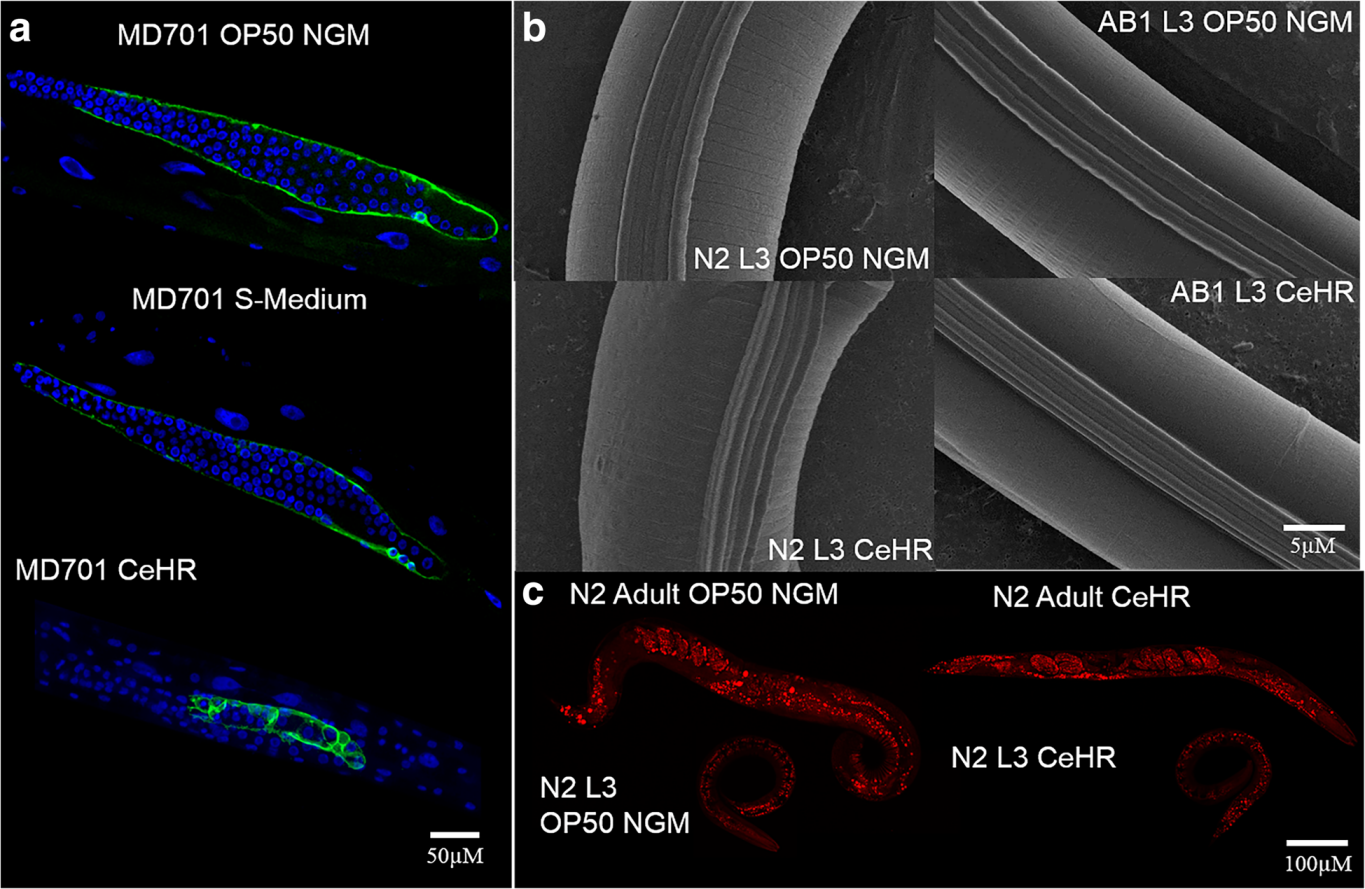
CeHR-grown *C. elegans* exhibit higher number of physiological germ cell apoptosis, more pronounced alae structures, and smaller fat stores. **a** OP50 NGM, S-Medium and CeHR grown MD701 adults were stain with DAPI. One or two germ cells enclosed by CED-1::GFP are detected around the loop region of the gonads in MD701 adults grown in OP50 NGM and S-Medium (84.6 and 100% of the worms, respectively). CED-1::GFP is detected around a large proportion of germ cells in CeHR grown MD701 adults (90.9% of the worms). **b** Scanning electron microscope (SEM) micrographs of OP50 NGM and CeHR grown N2 and AB1 L3 animals. N2 and AB1 L3 animals display normal alae and annuli with grown on OP50 NGM (81.3 and 85.7% of the worms, respectively). N2 but not AB1 L3 exhibit more pronounced alae in CeHR (72.2 and 12.5% for N2 and AB1, respectively). **c** Nile red staining of L3 and adult N2 *C. elegans* grown on OP50 NGM and CeHR. The number of fat stores is indistinguishable between OP50 NGM and CeHR grown N2 animals, but the fat stores significantly larger in OP50 NGM (81.8% large; 18.2% small) than CeHR raised N2 adults (27.3% large; 72.2% small) around the abdominal and tail regions

Then, we asked if these germline abnormalities were caused by an increase in physiological germ cell apoptosis. We utilized a transgenic line of *C. elegans* that expresses functional CED-1::GFP fusion protein in sheath cells [35]. CED-1 functions by initiating a signaling pathway in phagocytic cells that promotes cell corpse engulfment and is commonly used as a marker to detect cells undergoing the apoptotic pathway. In CeHR grown adults, CED-1::GFP was detected in a much higher proportion of germ cells (Fig. 6a, Additional file 1: Figure S8). Why the number of apoptotic germ cells in CeHR grown N2 adults were increased and whether or not this was the sole cause of aberrant germlines will require further investigations.

For the N2 worms, another GO enrichment term was collagen and cuticulin-based cuticle development for upregulated genes in CeHR compared to agar and reversion generations. Hence, we reasoned that the cuticles might show morphological differences between agar and CeHR-grown N2 animals. In adult animals, cuticular structures were difficult to distinguish between N2 and AB1 via scanning electron microscopy (SEM). More pronounced cuticular structures were detected in the earlier larval stages (L3) with SEM. We observed more protruding alae structures in CeHR-grown N2 larvae compared to AB1 larvae, but the annuli were indistinguishable between the two strains (Fig. 6b). Comparing 42 genes annotated with alae morphology variant from WormBase to our data, we identified 15 of these genes (*dpy-2*, *dpy-3*, *dpy-5*, *dpy-7*, *dpy-8*, *dpy-9*, *dpy-10*, *dpy-18*, *lon-3*, *qua-1*, *rol-6*, *sqt-1*, *sqt-2*, *sqt-3*, and *mlt-10*) upregulated under CeHR conditions in N2 but not in AB1 (Additional file 1: Figures S7 and S8). Taken together, the correlation between the function of the DEGs and the observed phenotype suggests that these genes can potentially be a part of underlying genetic networks causing these phenotypes.

Previously, we demonstrated that the CeHR-grown animals were thinner and longer than the OP50 NGM raised animals [6]. We asked if this change in body morphology was due to a reduction in fat stores that may have resulted from the absence of a bacterial diet. N2 animals grown on OP50 NGM and CeHR were stained with Nile Red [36] to visualize fat stores in all tissues of L3 and adult animals. We observed no significant difference in the number of fat stores between OP50 NGM and CeHR-grown N2 animals; however, larger fat stores were apparent in the abdominal and tail regions of OP50 NGM grown N2 adults (Fig. 6c, Additional file 1: Figure S8). Thus, the lack of a bacterial diet apparently contributes to the morphological differences through the fat storage.

## Discussion

In this paper, we demonstrated that environment changes cause significant alterations in the transcriptional responses in a *C. elegans* model. Different strains of the worms present unique transcriptional characteristics in the adaptive responses, and these characteristics show correlation with the observed phenotypes. For instance, we detected differential expression in the body morphology-related genes along with the phenotypical changes on the body morphology in CeHR-grown N2 strain, but not the AB1 strain. Furthermore, we found that different environments provoke the condition-specific expression of genes, and some of these genes lacked previous experimental EST or cDNA evidence.

Our results revealed that specific groups of genes are expressed only in particular environments. Some of these genes lacked transcript evidence previously (Fig. 1e). Considerable effort has been devoted to annotating the genes in *C. elegans*, but the standard WormBase [20] annotation still contains thousands of “predicted genes” (about 8%) which lack direct experimental cDNA or EST evidence [37]. The predicted genes in the *C. elegans* genome have been mainly identified by computational methods. Experimental detection of these genes was missing; mostly because they demonstrate poor expression, weak statistical signals or less conservation across species [38]. Others found that unused genetic information can remain in the genome for many generations [39]. Reactivation of dormant genes has been used for the treatment of particular disorders indicating that the silenced genes can have essential functions in the genome [40]. These studies provide evidence that dormant genes can be available on the genome throughout many generations and they can contain essential genetic information. Thus, we entertain the possibility that these predicted genes simply need a trigger to activate their expression, and this trigger may be elicited when animals are exposed to certain environmental stimuli.

A better understanding of the biological systems can be achieved through mining publicly available data sources [41]. We expanded our knowledge in the affected genetic systems by acquiring the ncRNA profiles from WormBase [20]. Condition-specific expression of ncRNAs implies important regulatory functions for these molecules in adaptive responses. Earlier studies have identified various classes of ncRNAs and their roles in biological systems including RNA splicing, DNA replication, epigenetic regulation of gene expression, and X-chromosome inactivation [42–46]. However, the majority of predicted ncRNAs have properties or functions that have not been identified yet. The list of unclassified ncRNAs, or “ncRNAs,” employed in this study was derived from the ‘7 k-set’ that was generated by the modENCODE Consortium [37]. The ‘7 k-set’ was assembled via predictions based on conservation and RNA secondary structure, and therefore, functional genomic studies of these “ncRNAs” are also still lacking. The co-enrichment of the ncRNA expressions with the coding ones suggest an interplay between these two.

In our RNA-seq experiments, we used poly(A) selection method but still observed numerous ncRNA molecules potentially because many eukaryotic ncRNAs are polyadenylated. For example, a poly(A) tail is part of the mature RNA for many long ncRNAs, i.e., *Xist* that mediates X-chromosome inactivation [47]. We cannot rule out that the ncRNAs sampled in our study resulted from technical artifacts in the RNA-seq. Nevertheless, many ncRNAs had expression levels higher than the housekeeping gene *pmp-3*. These findings would seem to suggest that the sampling of these RNAs was not an artifact of RNA-seq*.* The inclusion of non-polyadenylated ncRNA molecules can bring more insights for understanding the ncRNA functions in adaptive responses.

Along with the phenotypical differences, there were considerable differences in RNA expressions between the N2 and AB1 strains which were cultured in the same growth conditions. N2 had been reported to exhibit innate immune response against pathogenic bacteria [48, 49]. However, previous studies have determined that the N2 and wild-isolate CB4856 strains display a high variation in gene expressions and many of these strain-specific variations are related to innate immunity genes [50]. Similarly, our GO analysis results showed enrichment in innate immune response for the genes expressed exclusively in the AB1 strain on OP50 NGM (Fig. 2). These genes do not correspond to the previously reported divergent regions between the strains which might have caused the differences in gene expressions [51]. The AB1 animals are challenged more with pathogens in their natural habitat. The AB1-specific expression of innate immune response genes may indicate that the expression of these genes are more readily available in the AB1 strain than N2 to prepare the animals for a potential pathogen encounter. The results seem to suggest that *C. elegans* in the wild have the natural aptitude to survive not only on different food sources but also many other environmental variables including pathogens or the shifts between the solid and liquid settings. However, years of laboratory domestication of the N2 strain may have given rise to laboratory selections or genetic bottlenecks that may have hindered the ability of the N2 animals to acclimate to changing environments [14]. Alternatively, a potential presence of cis- and trans- eQTL between N2 and AB1 can be the underlying reason for the differences in the enrichment of innate response genes. Follow-up experiments are needed to determine the underlying reason for the expression differences in the innate immunity genes between N2 and the wild-isolates AB1 and CB4856.

For the majority of the *C. elegans* studies, the worms are cultured on bacteria-seeded agar plates instead of an axenic medium [13, 52]. However, a bacterial food source can present confounding effects on certain studies. For example, bacterial by-products can create genetic responses in the animals, and these responses are often overlooked. Axenic media is desirable in space, biochemical, and toxicology studies as it enables automated culturing and experimentation, and it eliminates the potential contamination risks due to a bacterial diet [6, 53, 54]. Placing the animals in axenic media, however, have profound consequences altering a variety of biological processes. To distinguish whether the genetic responses are from the case study or the axenic media conditions, a separate investigation should be conducted. In light of this, we revealed that *C. elegans* acquire large variations in gene expressions upon single generation exposure to two types of liquid media, and these variations are more prominent for the N2 strain.

## Conclusions

Culturing the worms in axenic medium seems to affect the worms more than only changing their physical environment to liquid in the S-Medium. Our results highlight that caution must be used while studying model organisms as the constant laboratory environments can cause substantial differences in the transcriptomic and phenotypic responses to abrupt environmental changes. We believe our data can provide standard controls for future studies that utilize liquid cultivation of *C. elegans* for experimentations and is a rich resource for the discovery of genes showing environment-specific expression.

## Methods

### *C. elegans* strains and growth conditions

Wild-type N2, wild-isolate AB1, and MD701 bcIs39 [lim-7p::ced-1::GFP + lin-15(+)] strains were obtained from the Caenorhabditis Genetics Center (CGC). All animals were grown at 21 °C. Embryos were isolated via the bleaching protocol in each step [52] and placed in the following growth media: *E. coli* OP50 seeded on NGM agar plates (OP50 NGM), 1 mL of S-Medium in 20 mL scintillation vials inoculated with a concentrated *E. coli* OP50 pellet made in 6 mL of an overnight culture (OP50 S-Medium), and CeHR medium in 20 mL scintillation vials according to Nass and Hamza [3] with minor modifications (we used 50 μM hemin instead of 20 μM and used 30 μM HEPES instead of 10 μM).

### RNA isolation, Illumina sequencing

Total RNA from approximately 100,000 synchronized young adult *C. elegans* (4 h post L4) were isolated with a modified TRIzol protocol and recovered by alcohol precipitation. Total RNA was further purified by PureLink ™ RNA Mini Kit (Life Technologies). Two micrograms of total RNA were used for library preparations using the TruSeq Stranded mRNA LT Sample Prep Kit (Illumina). Libraries were sequenced on an Illumina HiSeq 2500 instrument set to the rapid run mode using single-end, 1 × 51 cycle sequencing reads as per manufacturer’s instructions.

### qRT-PCR

Quantitative RT-PCR was conducted using 1st strand cDNA synthesized from total RNA and gene-specific primers (Additional file 1: Table S3). Each cDNA sample was amplified using the SYBR® Premix Ex Taq ™ II (Takara Bio) on the ABI 7500 Fast Real-time PCR System (Applied Biosystem) according to the manufacturers. The experiment was performed by three independent experiments with biological triplicates.

### Bioinformatics analyses

The Tuxedo pipeline [55] was used to find DEGs with default parameters. The reference genome (WBCel235) was obtained from Ensembl [56]. Data from the study conducted by Dallaire et al. were retrieved from Gene Expression Omnibus (accession number GSE54173) [21] to compare the expression of the unconfirmed genes in OP50 NGM-grown N2, and the same pipeline with the same parameters were used for the analysis of the data. The same pipeline with same parameters were used to analyze RNA-seq data. We considered genes with FPKM > 1, FDR adjusted *p-values* < *0.05*, and log_2_ fold change > 2 as differentially expressed. miRNA, piRNA, and rRNA molecules were discarded from the analysis. The ncRNA molecules (WS250) and unconfirmed genes were acquired from WormBase through the ftp site and personal communications, respectively [20]. The motif enrichment analysis was performed with MEME Suite (v4.11.1) [32]. The promoter sequences for the motif enrichment were retrieved from UCSC Genome Browser database for ce11 [57]. Find Individual Motif Occurrences (FIMO) was used to find the enrichment of the detected sequence motifs for the known transcription factor recognition sites [58]. We utilized GOMO (v4.12.0) software to identify the enriched human GO terms for the promoter sequence motifs [59]. GO and pathway enrichment analyses was made with the Database for Annotation, Visualization and Integrated Discovery (DAVID) (v6.8) [60], and Benjamini Hochberg corrected and FDR adj. *p-values* < 0.05 considered significant. Tissue and phenotype enrichment analyses were made by using WormBase Gene Set Enrichment Analysis tool [61], FDR adj. *p-values* < 0.05 considered significant. Long, thin, and alae morphology variant phenotype genes were retrieved from WormBase (WS262). R software was used for all statistical analyses and the plots [62].

### Microscopy

Nematodes were fixed in cold (− 20 °C) methanol for 15 min and stained with SYBR Gold (1:500,000 dilution in PBS) or DAPI (100 ng/mL) for 15 min, or Nile Red (5 ng/mL) for 30 min, at 21 °C with gentle rocking. Worms were destained in PBST, washed three times with M9 and mounted on a 2% agarose pad for microscopy. All micrographs were taken with the Zeiss 710 laser scanning confocal microscope with a 40×/1.2 N.A. water immersion objective. Dissected gonads were stained with SYBR gold as mentioned above. Nematodes were placed onto 0.22 μM filters where the excess liquid was removed to visualize cuticular structures via cryo-SEM. Upon attaching the filter paper onto the sample holder, samples were plunged into liquid nitrogen slush. There a vacuum was pulled allowing sample transfer to the Gatan Alto 2500 cryo chamber at a temperature of − 125 °C. Samples were sublimated for 10 min at 90 °C followed by cooling to − 125 °C. A thin layer of Gold Palladium was sputtered onto the samples. The samples were then transferred into a Hitachi S-4700 Field-Emission Scanning Electron Microscope for imaging. At least 20 animals were screened for each phenotype, and the represented figures were used for the observed phenotypes.

## Additional files

Additional file 1: Effects of liquid cultivation on gene expression and phenotype of *C. elegans*. (PDF 1780 kb)

Additional file 2: Commonly expressed ncRNA molecules in the three experiments. (TXT 6 kb)

Additional file 3: Genotype, environment, and genotype-environment interaction induced genes. (XLSX 5595 kb)

## Abbreviations

CeHR: *C. elegans* Habitation and Reproduction
CeMM: *C. elegans* Maintenance Medium
DAVID: Database for Annotation, Visualization and Integrated Discovery
DEG: Differentially expressed gene
EST: Expressed sequence tag
FDR: False discovery rate
FIMO: Find Individual Motif Occurrences
FPKM: Fragments Per Kilobase of transcript per Million mapped reads
GO: Gene ontology
ncRNA: Non-coding RNA
NGM: Nematode growth medium
RNA-seq: RNA-sequencing

## References

[1] Pierce-Shimomura JT, Chen BL, Mun JJ, Ho R, Sarkis R, SL MI (2008). Genetic analysis of crawling and swimming locomotory patterns in *C. elegans*. Proc. Natl. Acad. Sci.

[2] Szewczyk NJ, Kozak E, Conley CA. Chemically defined medium and *Caenorhabditis elegans*. BMC Biotechnol. 2003 ;3:19.[cited 2015 Aug 27] Available from: https://www.ncbi.nlm.nih.gov/pmc/articles/PMC270041/?tool=pmcentrez&report=abstract. 10.1186/1472-6750-3-19 PMC270041 14580264

[3] Nass R, Hamza I. The nematode C. elegans as an animal model to explore toxicology in vivo: solid and axenic growth culture conditions and compound exposure parameters. Curr. Protoc. Toxicol. 2007;Chapter 1:Unit1.9. 1–9. 10.1002/0471140856.tx0109s31 20922756

[4] Houthoofd K, Braeckman BP, Lenaerts I, Brys K, De Vreese A, Van Eygen S, et al. Axenic growth up-regulates mass-specific metabolic rate, stress resistance, and extends life span in *Caenorhabditis elegans*. Exp. Gerontol. 2002 ;37:1371–1378. [cited 2015 Aug 27] Available from: http://www.sciencedirect.com/science/article/pii/S0531556502001730. 10.1016/s0531-5565(02)00173-0 12559406

[5] Szewczyk NJ, Udranszky IA, Kozak E, Sunga J, Kim SK, Jacobson LA, et al. Delayed development and lifespan extension as features of metabolic lifestyle alteration in *C. elegans* under dietary restriction. J. Exp. Biol. 2006 ;209:4129–4139. [cited 2015 Aug 27] Available from: http://jeb.biologists.org/content/209/20/4129.full. 10.1242/jeb.02492 17023606

[6] Doh JH, Moore AB, Çelen İ, Moore MT, Sabanayagam CR (2016). ChIP and Chips : Introducing the WormPharm for correlative studies employing pharmacology and genome-wide analyses in C . elegans. J Biol Methods.

[7] Daxinger L, Whitelaw E. Transgenerational epigenetic inheritance: more questions than answers. Genome Res. 2010 ;20:1623–1628. [cited 2015 Jul 30] Available from: http://www.pubmedcentral.nih.gov/articlerender.fcgi?artid=2989988&tool=pmcentrez&rendertype=abstract. 10.1101/gr.106138.110 PMC2989988 21041414

[8] Jaenisch R, Bird A (2003). Epigenetic regulation of gene expression: how the genome integrates intrinsic and environmental signals. Nat. Genet.

[9] Heard E, Martienssen RA. Transgenerational epigenetic inheritance: myths and mechanisms. Cell. 2014;157:95–109. [cited 2014 Jul 10] Available from: http://www.sciencedirect.com/science/article/pii/S0092867414002864. 10.1016/j.cell.2014.02.045 PMC4020004 24679529

[10] Selch F, Higashibata A, Imamizo-Sato M, Higashitani A, Ishioka N, Szewczyk NJ, et al. Genomic response of the nematode *Caenorhabditis elegans* to spaceflight. Adv. Space Res. 2008;41:807–815. [cited 2015 Oct 22] Available from: http://www.pubmedcentral.nih.gov/articlerender.fcgi?artid=2288577&tool=pmcentrez&rendertype=abstract. 10.1016/j.asr.2007.11.015 PMC2288577 18392117

[11] Félix M-A, Braendle C (2010). The natural history of Caenorhabditis elegans. Curr Biol.

[12] Brenner S (1974). The genetics of Caenorhabditis elegans. Genetics.

[13] Stiernagle T. Maintenance of *C. elegans*. WormBook; 2006 1–11. [cited 2015 Apr 27]; Available from: http://www.ncbi.nlm.nih.gov/books/NBK19649/. 10.1895/wormbook.1.101.1 PMC4781397 18050451

[14] Sterken MG, Snoek LB, Kammenga JE, Andersen EC. The laboratory domestication of Caenorhabditis elegans. Trends Genet. 2015;31:224–31. 10.1016/j.tig.2015.02.009 PMC4417040 25804345

[15] Barrière A, Félix M-A, Barriere A, Felix M-A, Barrière A, Félix M-A, et al. Natural variation and population genetics of Caenorhabditis elegans. WormBook. 2005:1–19. 10.1895/wormbook.1.43.1 PMC4781346 18050391

[16] Kramer M, Kranz AL, Su A, Winterkorn LH, Albritton SE, Ercan S. Developmental dynamics of X-chromosome dosage compensation by the DCC and H4K20me1 in C. Elegans. PLoS Geenet 2015;11. 10.1371/journal.pgen.1005698 PMC4671695 26641248

[17] Blazie SM, Babb C, Wilky H, Rawls A, Park JG, Mangone M. Comparative RNA-Seq analysis reveals pervasive tissue-specific alternative polyadenylation in Caenorhabditis elegans intestine and muscles. BMC Biol 2015;13. 10.1186/s12915-015-0116-6 PMC4343181 25601023

[18] Prabh N, Rödelsperger C. Are orphan genes protein-coding, prediction artifacts, or non-coding RNAs? BMC Bioinformatics 2016;17. 10.1186/s12859-016-1102-x PMC4888513 27245157

[19] Duff MO, Olson S, Wei X, Garrett SC, Osman A, Bolisetty M (2015). Genome-wide identification of zero nucleotide recursive splicing in drosophila. Nature.

[20] Harris TW, Baran J, Bieri T, Cabunoc A, Chan J, Chen WJ, et al. WormBase 2014: new views of curated biology. Nucleic Acids Res. 2014;42:D789–D793.[cited 2015 Jul 6] Available from: http://nar.oxfordjournals.org/content/42/D1/D789. 10.1093/nar/gkt1063 PMC3965043 24194605

[21] Dallaire A, Proulx S, Simard MJ, Lebel M. Expression profile of Caenorhabditis elegans mutant for the Werner syndrome gene ortholog reveals the impact of vitamin C on development to increase life span. BMC Genomics 2014;15:940. 10.1186/1471-2164-15-940 PMC4221712 25346348

[22] Hoogewijs D, Houthoofd K, Matthijssens F, Vandesompele J, Vanfleteren JR (2008). Selection and validation of a set of reliable reference genes for quantitative sod gene expression analysis in *C. elegans*. BMC Mol. Biol.

[23] Zhang Y, Chen D, Smith MA, Zhang B, Pan X. Selection of reliable reference genes in caenorhabditis elegans for analysis of nanotoxicity. PLoS One 2012;7:e31849. 10.1371/journal.pone.0031849 PMC3305280 22438870

[24] Li C, Kim K. Neuropeptides. WormBook. 2008;1–36. Available from: http://www.pubmedcentral.nih.gov/articlerender.fcgi?artid=2749236&tool=pmcentrez&rendertype=abstract. 10.1895/wormbook.1.142.1 PMC2749236 18819171

[25] López-Maury L, Marguerat S, Bähler J (2008). Tuning gene expression to changing environments: from rapid responses to evolutionary adaptation. Nat Rev Genet [Internet].

[26] Gems D, Riddle DL. Genetic, behavioral and environmental determinants of male longevity in *Caenorhabditis elegans*. Genetics. 2000 ;154:1597–1610. [cited 2017 Jul 17] Available from: http://www.ncbi.nlm.nih.gov/pubmed/10747056. 10.1093/genetics/154.4.1597 PMC1461011 10747056

[27] Croll NA, Smith JM, Zuckerman BM. The aging process of the nematode Caenorhabditis elegans in bacterial and axenic culture. Exp. Aging Res. 1977 ;3:175–189.[cited 2017 Jul 17] Available from: http://www.ncbi.nlm.nih.gov/pubmed/334555. 10.1080/03610737708257101 334555

[28] Castelein N, Hoogewijs D, De Vreese A, Braeckman BP, Vanfleteren JR (2008). Dietary restriction by growth in axenic medium induces discrete changes in the transcriptional output of genes involved in energy metabolism in Caenorhabditis elegans. Biotechnol J.

[29] Zhao S, Fung-Leung WP, Bittner A, Ngo K, Liu X. Comparison of RNA-Seq and microarray in transcriptome profiling of activated T cells. PLoS One 2014;9:e78644. 10.1371/journal.pone.0078644 PMC3894192 24454679

[30] Narasimhan SD, Yen K, Tissenbaum HA. Converging pathways in lifespan regulation. Curr Biol. 2009;19(15):R657–66. 10.1016/j.cub.2009.06.013 PMC3109866 19674551

[31] Hibshman JD, Hung A, Baugh LR. Maternal diet and insulin-like signaling control intergenerational plasticity of progeny size and starvation resistance. PLoS Genet 2016;12:e1006396. 10.1371/journal.pgen.1006396 PMC5081166 27783623

[32] Bailey TL, Boden M, Buske FA, Frith M, Grant CE, Clementi L, et al. MEME suite: tools for motif discovery and searching. Nucleic Acids Res 2009;37:W202–8. 10.1093/nar/gkp335 PMC2703892 19458158

[33] Riesen M, Feyst I, Rattanavirotkul N, Ezcurra M, Tullet JMA, Papatheodorou I, et al. MDL-1, a growth- and tumor-suppressor, slows aging and prevents germline hyperplasia and hypertrophy in &lt;i&gt;C. elegans&lt;/i&gt; Aging (Albany. NY). 2014 ;6:98–117. [cited 2018 Feb 21]Available from: http://www.ncbi.nlm.nih.gov/pubmed/24531613. 10.18632/aging.100638 PMC3969279 24531613

[34] Reinke V, Krause M, Okkema P. Transcriptional regulation of gene expression in *C. elegans*. WormBook. 2013;1–34. Available from: http://www.pubmedcentral.nih.gov/articlerender.fcgi?artid=3893038&tool=pmcentrez&rendertype=abstract%5Cn, http://www.ncbi.nlm.nih.gov/pmc/articles/PMC3893038/%5Cn, http://www.ncbi.nlm.nih.gov/pubmed/23801596%5Cn, http://www.pubmedcentral.nih.gov/articlerender.f. 10.1895/wormbook.1.45.2 PMC3893038 23801596

[35] Zhou Z, Hartwieg E, Horvitz HR (2001). CED-1 is a transmembrane receptor that mediates cell corpse engulfment in C. Elegans. Cell.

[36] Greenspan P, Mayer EP, Fowler SD (1985). Nile red: a selective fluorescent stain for intracellular lipid droplets. J Cell Biol.

[37] Gerstein MB, Lu ZJ, Van Nostrand EL, Cheng C, Arshinoff BI, Liu T, et al. Integrative analysis of the *Caenorhabditis elegans* genome by the modENCODE project. Science 2010 ;330:1775–1787. [cited 2015 Oct 29] Available from: http://www.sciencemag.org/content/330/6012/1775.abstract. 10.1126/science.1196914 PMC3142569 21177976

[38] Hillier LW, Coulson A, Murray JI, Bao Z, Sulston JE, Waterston RH. Genomics in *C. elegans*: so many genes, such a little worm. Genome Res. 2005 ;15:1651–1660. [cited 2016 May 13] Available from: http://genome.cshlp.org/content/15/12/1651.full. 10.1101/gr.3729105 16339362

[39] Collin R, Cipriani R (2003). Dollo’s law and the re-evolution of shell coiling. Proc R Soc B Biol Sci [Internet].

[40] Huang H-S, Allen JA, Mabb AM, King IF, Miriyala J, Taylor-Blake B (2011). Topoisomerase inhibitors unsilence the dormant allele of Ube3a in neurons. Nature [Internet].

[41] Çelen İ, Ross KE, Arighi CN, Wu CH. Bioinformatics Knowledge Map for Analysis of Beta-Catenin Function in Cancer. PLoS One . Public Library of Science; 2015 [cited 2];10:e0141773. Available from: http://journals.plos.org/plosone/article?id=10.1371/journal.pone.0141773. 10.1371/journal.pone.0141773 PMC4624812 26509276

[42] Carthew RW, Sontheimer EJ. Origins and Mechanisms of miRNAs and siRNAs. Cell. 2009. p. 642–55. 10.1016/j.cell.2009.01.035 PMC2675692 19239886

[43] Kaikkonen MU, Lam MTY, Glass CK. Non-coding RNAs as regulators of gene expression and epigenetics. Cardiovasc. Res. 2011. p. 430–40. 10.1093/cvr/cvr097 PMC3096308 21558279

[44] Kishore S (2006). The snoRNA HBII-52 Regulates Alternative Splicing of the Serotonin Receptor 2C. Science.

[45] Zhang AT, Langley AR, Christov CP, Kheir E, Shafee T, Gardiner TJ (2011). Dynamic interaction of Y RNAs with chromatin and initiation proteins during human DNA replication. J Cell Sc.

[46] Cao J (2014). The functional role of long non-coding RNAs and epigenetics. Biol. Proced. Online.

[47] Amaral PP, Mattick JS. Noncoding RNA in development. Mamm. Genome. 2008. p. 454–92. 10.1007/s00335-008-9136-7 18839252

[48] Alper S, McBride SJ, Lackford B, Freedman JH, Schwartz DA (2007). Specificity and complexity of the Caenorhabditis elegans innate immune response. Mol Cell Biol [Internet].

[49] Battisti JM, Watson LA, Naung MT, Drobish AM, Voronina E, Minnick MF. Analysis of the Caenorhabditis elegans innate immune response to Coxiella burnetii. Innate Immun. [internet]. 2016;1753425916679255. Available from: http://www.ncbi.nlm.nih.gov/pubmed/27884946. 10.1177/1753425916679255 PMC5266666 27884946

[50] Capra EJ, Skrovanek SM, Kruglyak L (2008). Comparative developmental expression profiling of two *C. elegans* isolates. PLoS One.

[51] Thompson OA, Snoek LB, Nijveen H, Sterken MG, Volkers RJM, Brenchley R, Van't Hof A, et al. Remarkably divergent regions punctuate the genome assembly of the Caenorhabditis elegans Hawaiian strain CB4856. Genet. 2015;genetics-115. 10.1534/genetics.115.175950 PMC4512556 25995208

[52] Sulston J, Hodgkin J, Wood WB (1988). The Nematode *Caenorhabditis elegans*.

[53] Adenle AA, Johnsen B, Szewczyk NJ. Review of the results from the International *C. elegans* first experiment (ICE-FIRST). Adv Sp Res. 2009;44:210–16. 10.1016/j.asr.2009.04.008 PMC2719817 20161164

[54] Samuel TK, Sinclair JW, Pinter KL, Hamza I. Culturing *Caenorhabditis elegans* in axenic liquid media and creation of transgenic worms by microparticle bombardment. J. Vis. Exp. [Internet]. 2014 [cited 2017 Jun 2];e51796. Available from: http://www.jove.com/video/51796/culturing-caenorhabditis-elegans-axenic-liquid-media-creation. 10.3791/51796 PMC4500161 25145601

[55] Trapnell C, Roberts A, Goff L, Pertea G, Kim D, Kelley DR, et al. Differential gene and transcript expression analysis of RNA-seq experiments with TopHat and Cufflinks. Nat. Protoc. [Internet]. Nature Publishing Group, a division of Macmillan Publishers Limited. All Rights Reserved.; 2012 [cited 2014 Jul 9];7:562–78. Available from: 10.1038/nprot.2012.016. 10.1038/nprot.2012.016 PMC3334321 22383036

[56] Cunningham F, Amode MR, Barrell D, Beal K, Billis K, Brent S, et al. Ensembl 2015. Nucleic Acids Res. 2014 ;43:D662–D669. [cited 2014 Nov 25] Available from: http://nar.oxfordjournals.org/content/43/D1/D662. 10.1093/nar/gku1010 PMC4383879 25352552

[57] Rosenbloom KR, Armstrong J, Barber GP, Casper J, Clawson H, Diekhans M (2015). The UCSC genome browser database: 2015 update. Nucleic Acids Res.

[58] Grant CE, Bailey TL, Noble WS (2011). FIMO: scanning for occurrences of a given motif. Bioinformatics.

[59] Buske FA, Bodén M, Bauer DC, Bailey TL (2010). Assigning roles to DNA regulatory motifs using comparative genomics. Bioinformatics.

[60] Huang DW, Sherman BT, Lempicki RA (2008). Systematic and integrative analysis of large gene lists using DAVID bioinformatics resources. Nat Protoc.

[61] Angeles-Albores D, N. Lee RY, Chan J, Sternberg PW. Tissue enrichment analysis for C. elegans genomics. BMC Bioinformatics. 2016 ;17:366. [cited 2017 Jul 17] Available from: http://www.ncbi.nlm.nih.gov/pubmed/27618863. 10.1186/s12859-016-1229-9 PMC5020436 27618863

[62] R Development Core Team. R: A language and environment for statistical computing. R Foundation for Statistical Computing, Vienna, Austria. URL http://www.R-project.org/. R Found. Stat. Comput. Vienna, Austria. 2017.

