# Supplementary material for: Effects of liquid cultivation on gene expression and phenotype of C. elegans
Source: BMC Genomics. 2018 Jul 31;19:562. doi: 10.1186/s12864-018-4948-7 (PMC6069985; doi:10.1186/s12864-018-4948-7)
Supplement: Supplementary file 1 — Effects of liquid cultivation on gene expression and phenotype of C. elegans. (PDF 1780 kb) [file 12864_2018_4948_MOESM1_ESM.pdf]

Effects of liquid cultivation on gene expression and phenotype of *C. elegans*

İrem Çelen[a,b], Jung H. Doh[b], and Chandran R. Sabanayagam[b,1]

[a]Center for Bioinformatics and Computational Biology, University of Delaware, Newark, DE, USA, 19711

[b]Delaware Biotechnology Institute, University of Delaware, Newark, DE, USA, 19711

[1]Corresponding author:
Chandran R. Sabanayagam
Delaware Biotechnology Institute
University of Delaware
15 Innovation Way, Newark, DE 19711
Telephone: (302) 831-1691
E-mail: chandran@udel.edu

Contact information of authors:
İrem Çelen : irem@udel.edu
Jung H. Doh: jdoh@udel.edu
Chandran R. Sabanayagam: chandran@udel.edu

Keywords: Dormant genes, ncRNA, adaptation, reaction norm

Supplementary Figures

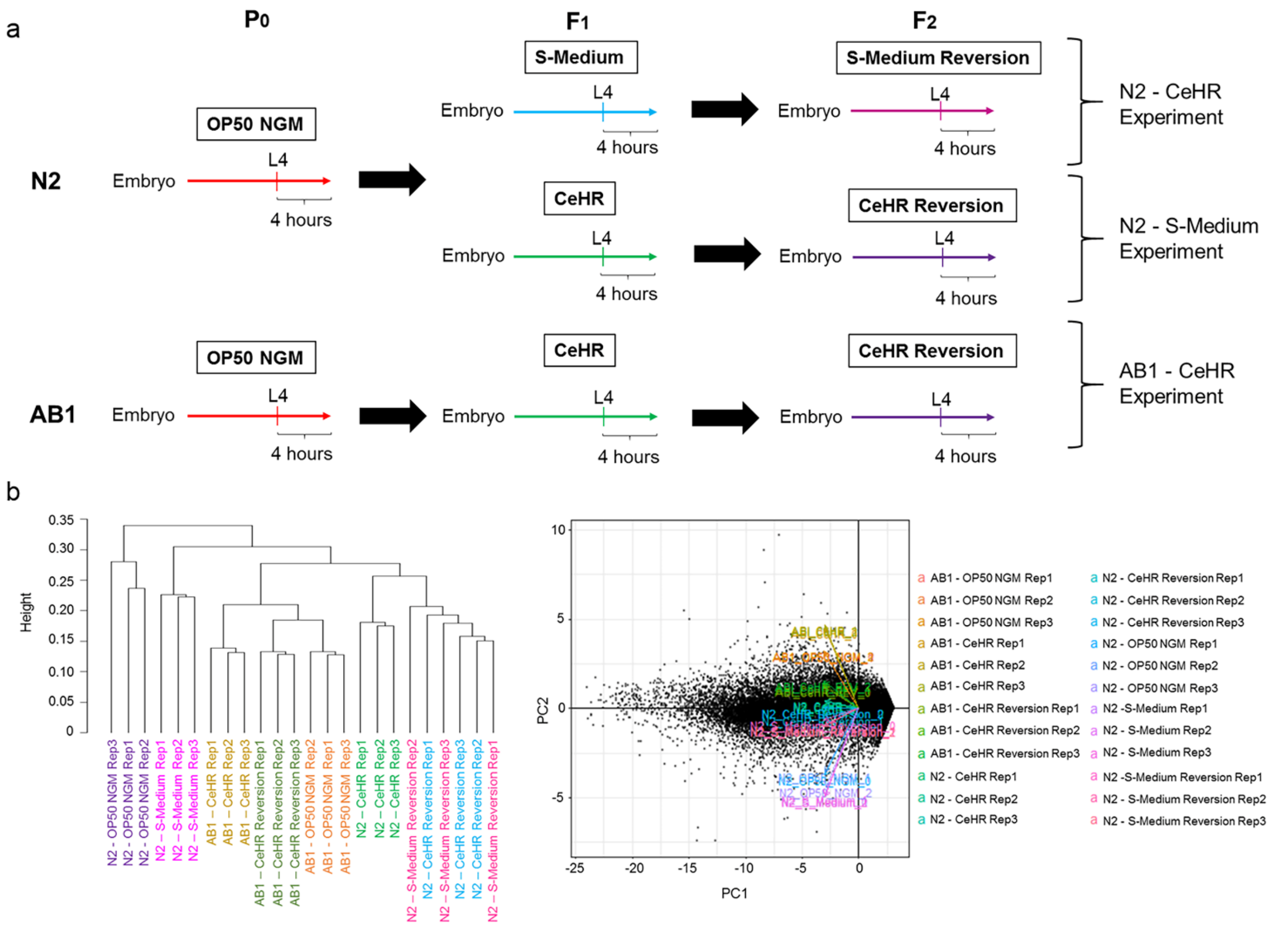

Fig. S1: Exposure of the domesticated and wild-isolate worms to environmental changes to induce a transcriptomic response. **(a)** Experimental design for multigenerational studies in N2 and AB1 strains of *C. elegans*. **(b)** Dendrogram and principal component analyses show a clear separation between the laboratory domesticated strain and the wild-isolate. Exposure of the N2 strain to environmental changes results in highly variant transcriptomic responses in comparison to the ones cultured on agar plates.

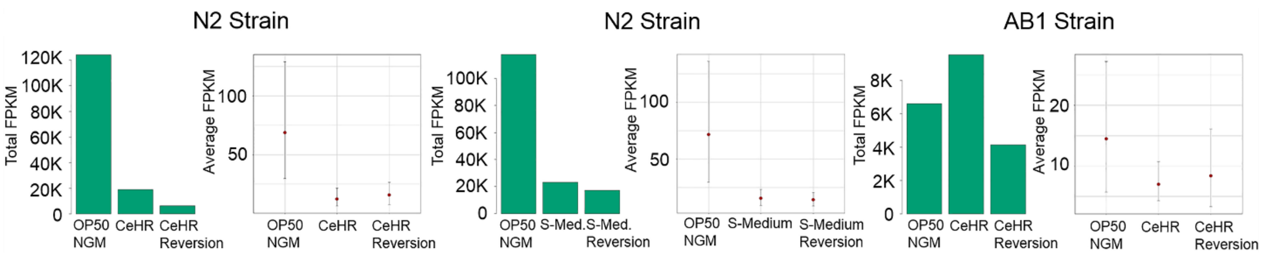

Fig. S2: The gene expression trends in response to environmental changes vary between the strains N2 strain shows an overall decrease in transcripts when exposed to environmental changes.

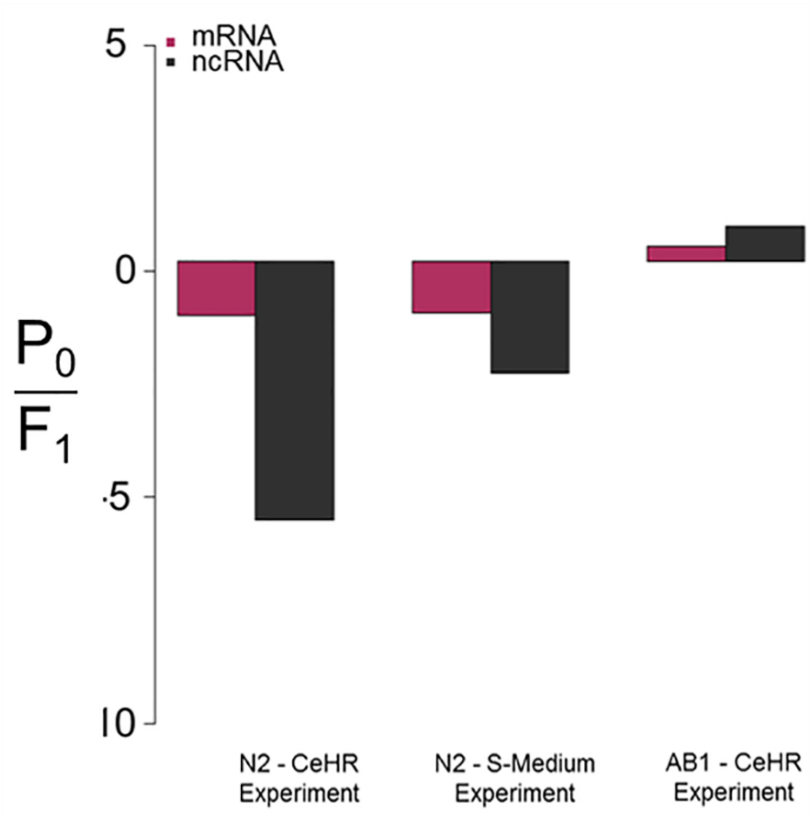

Fig. S3: The ratio of the expressed mRNA and ncRNA molecules between the P_0 and F_1 generations. Both the molecules in the N2 strain show a decrease in response to the environmental changes. In comparison to the decrease in mRNA levels, the proportion of expressed ncRNA molecules are lower under exposure to environmental changes.

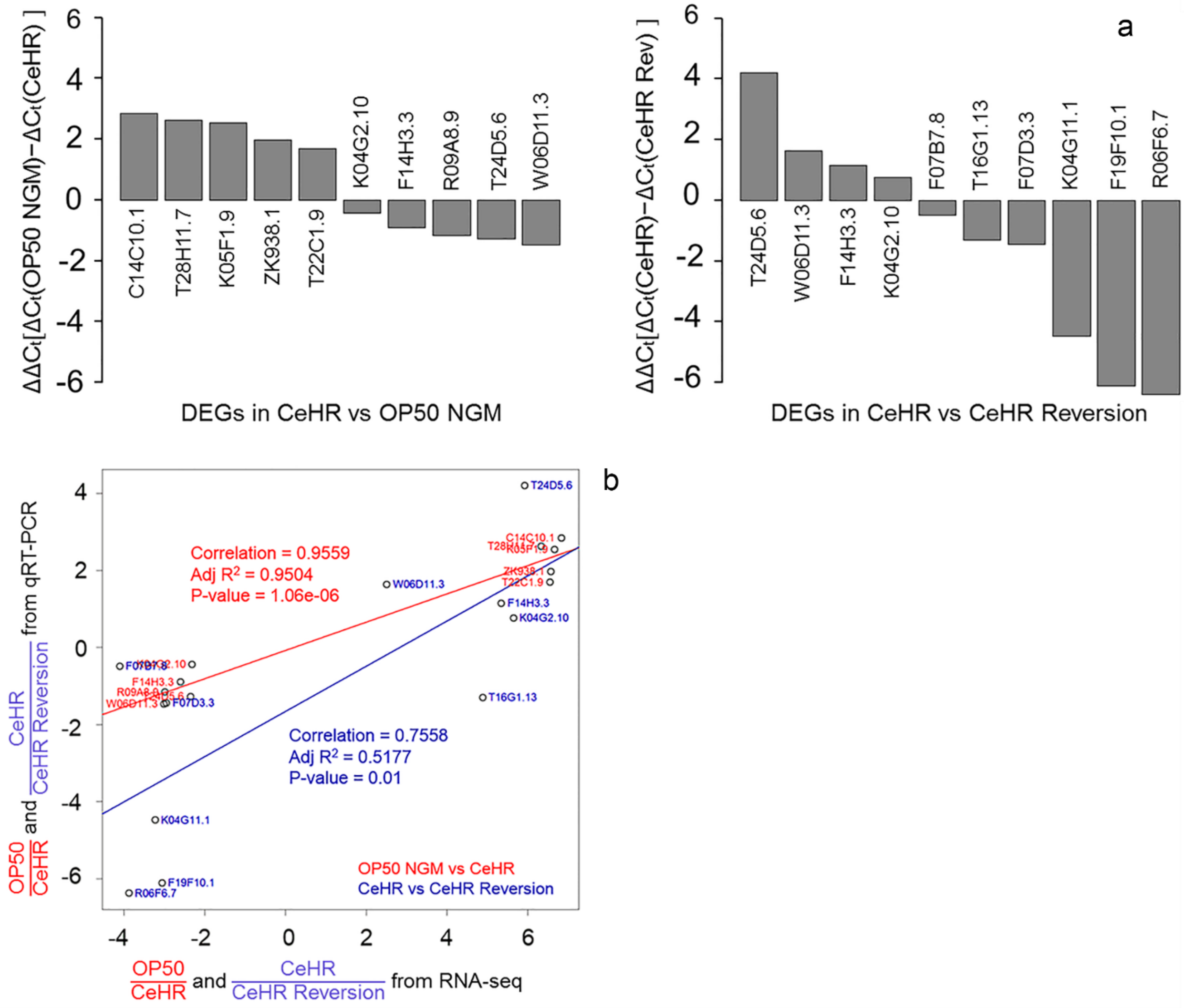

Fig. S4: Quantitative Real Time PCR (qRT-PCR) Analysis of differentially expressed RNA. **(a)** qRT-PCR were conducted with total RNA extracted from N2 animals in grown on OP50 NGM, CeHR and CeHR Reversion (back onto OP50 NGM). All genes, with the exception of T16G1.13, analyzed via qRT-PCR were consistent with the RNA-seq data and analysis of the differential gene expression. **(b)** Pearson correlation of the qRT-PCR and RNA-seq data.

a

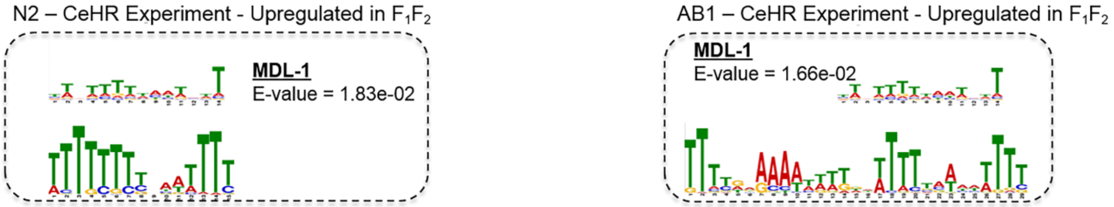

N2 – CeHR Experiment - Upregulated in F_1F_2

GO term (Human)	P-value	Specificity
Sensory perception of smell	8.479e-08	~88%
DNA damage checkpoint	1.111e-05	~77%
Transcription initiation from RNA polymerase II promoter	1.450e-05	~97%

AB1 – CeHR Experiment - Upregulated in F_1F_2

GO term (Human)	P-value	Specificity
Sensory perception of smell	8.479e-08	~88%
G-protein coupled receptor protein signaling pathway	1.696e-07	~7%
Positive regulation of immune response	1.221e-05	~6%

b

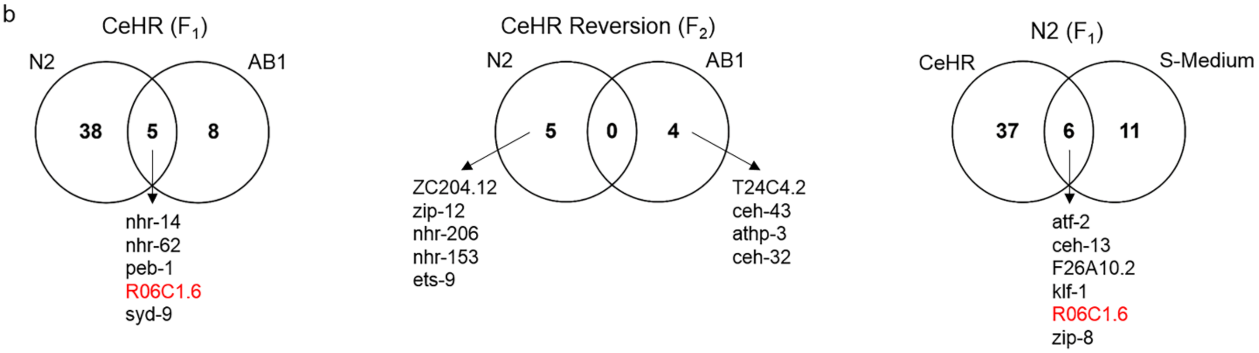

Fig. S5: Putative transcription factors functioning in response to environmental changes in N2 and AB1. **(a)** One motif from each strain showed enrichment as a recognition site for transcription factor MDL-1. The same sequence motifs demonstrated differences for the overrepresented biological functions in human. **(b)** Venn diagrams depicting the overlap of the differentially expressed transcription factors between environmental conditions or strains. The overlap between the F1 generations are statistically significant (Hypergeometric test, *P-value* < 0.0001)

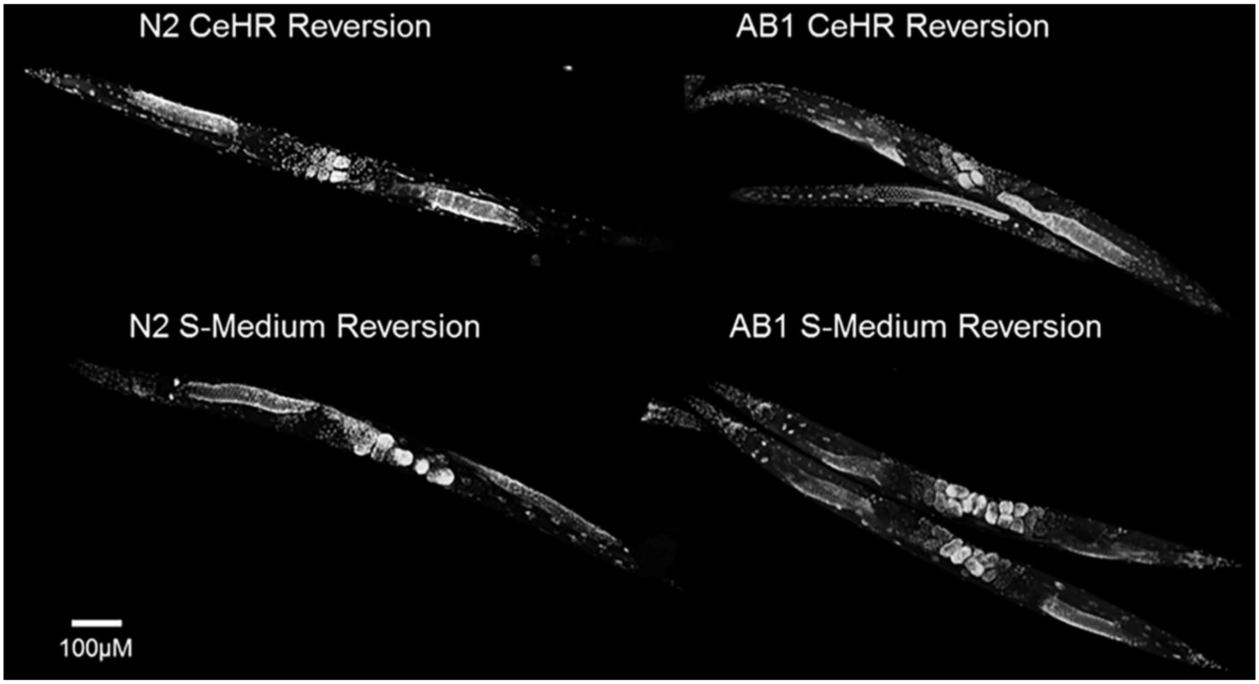

Fig. S6: F2 N2 and AB1 C. elegans that were reverted back onto OP50 NGM exhibit normal germ lines. N2 and AB1 animals were placed back onto OP50 NGM agar plates from CeHR or S-Medium. Adult hermaphrodites were stained with DAPI to visualize germ cell nuclei. All germ line exhibit normal germ cell morphologies and number.

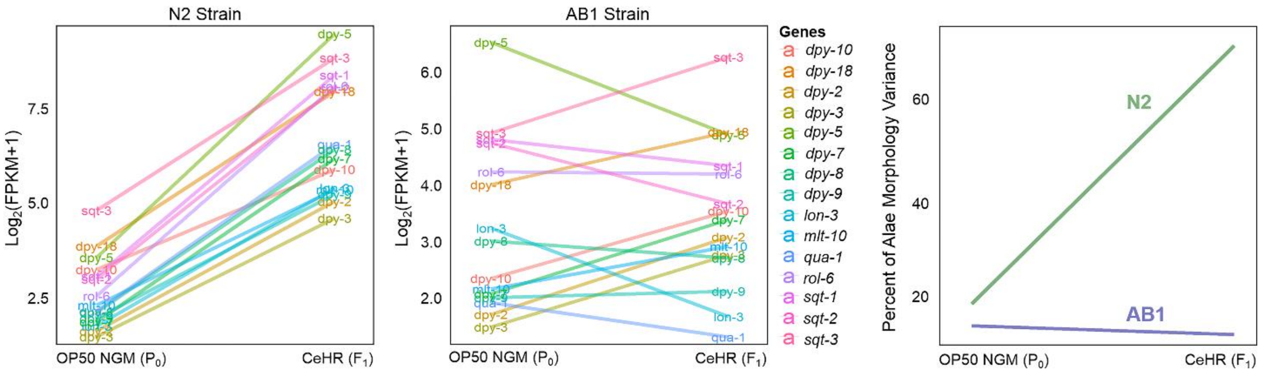

Fig. S7: Norm of reaction between the alae morphology variant annotated genes and the detected alae morphology variance between AB1 and N2 indicating a potential role for these genes in the observed phenotype.

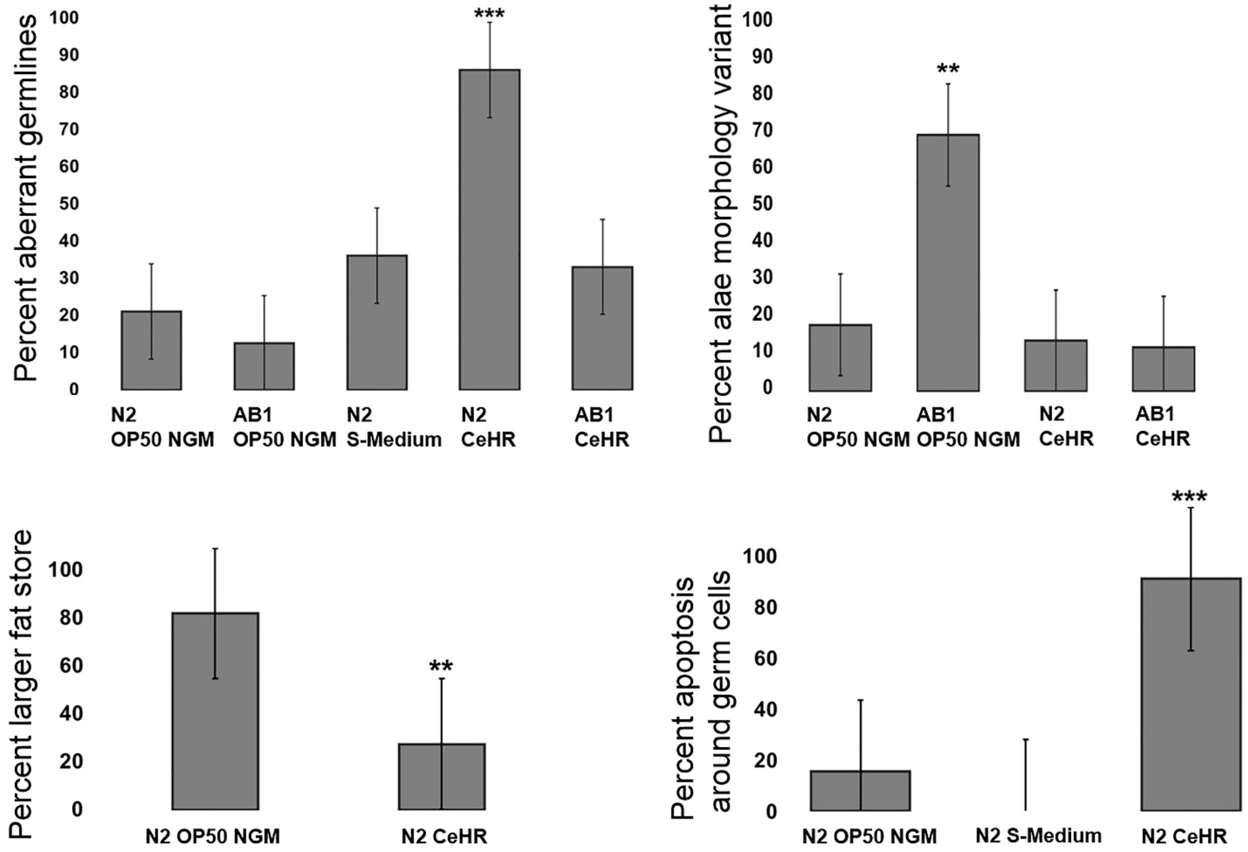

Fig. S8: Quantitative differences in the observed phenotypes in liquid cultivations. T-test was used to compare the conditions. Error bars represent the standard error. **: *P-value* < 0.01, ***: *P-value* < 0.0001. *P-value* > 0.05 is not reported.

Supplementary Tables

Table S1: Sequencing read information for the RNA-seq libraries

Strain	Condition	Replicate	Generation	Stage	Number of Reads	Mapped Reads
N2	Agar (OP50 NGM)	1	P0	Young adult	19,785,469	19,033,093 (96.2%)
N2	Agar (OP50 NGM)	2	P0	Young adult	17,804,139	17,078,192 (95.9%)
N2	Agar (OP50 NGM)	3	P0	Young adult	17,584,017	16,792,356 (95.5%)
N2	CeHR	1	F1	Young adult	29,616,506	27,904,230 (94.2%)
N2	CeHR	2	F1	Young adult	30,144,650	28,728,679 (95.3%)
N2	CeHR	3	F1	Young adult	29,749,460	27,582,792 (92.7%)
N2	CeHR Reversion	1	F2	Young adult	25,771,272	24,627,700 (95.6%)
N2	CeHR Reversion	2	F2	Young adult	49,360,215	46,951,509 (95.1%)
N2	CeHR Reversion	3	F2	Young adult	6,524,144	6,256,402 (95.9%)
N2	S-Medium	1	F1	Young adult	24,031,853	22,294,902 (92.8%)
N2	S-Medium	2	F1	Young adult	25,924,449	22,848,853 (88.1%)
N2	S-Medium	3	F1	Young adult	26,580,009	19,716,312 (74.2%)
N2	S-Medium Reversion	1	F2	Young adult	21,340,354	20,685,738 (96.9%)
N2	S-Medium Reversion	2	F2	Young adult	2,824,111	2,727,187 (96.6%)
N2	S-Medium Reversion	3	F2	Young adult	49,930,813	47,900,763 (95.9%)
AB1	Agar (OP50 NGM)	1	P0	Young adult	21,217,270	20,230,031 (95.3%)
AB1	Agar (OP50 NGM)	2	P0	Young adult	16,180,777	15,393,012 (95.1%)
AB1	Agar (OP50 NGM)	3	P0	Young adult	25,889,871	24,612,562 (95.1%)
AB1	CeHR	1	F1	Young adult	15,358,876	14,477,230 (94.3%)

Table S1 Cont.

AB1	CeHR	2	F1	Young adult	15,636,461	14,762,856 (94.4%)
AB1	CeHR	3	F1	Young adult	14,469,569	13,649,882 (94.3%)
AB1	CeHR Reversion	1	F2	Young adult	16,467,847	15,708,866 (95.4%)
AB1	CeHR Reversion	2	F2	Young adult	17,444,758	16,618,698 (95.3%)
AB1	CeHR Reversion	3	F2	Young adult	22,730,048	21,629,136 (95.2%)

Table S2: Raw data from the qRT-PCR analysis.

Condition Being Compared	Gene	Control Ct Ave (W)	Gene Ct Ave (X)	ΔCt Ave [X – W] = Y	ΔΔCt Agar(1]	CeHR	Agar	log2 (fold change)	test_stat	p-value	q-value	significant
Agar Vs CeHR	C14C10.1	18.5907 Ref in Agar	28.312	9.7209	2.8454	53.43	0.79	-6.0817	-9.74	5.00E-05	0.0001	yes
	ZK938.1		26.662	8.0712	1.9718	57.85	0.94	-5.94209	-10.9	0.00005	0.0001	yes
	K05F1.9		28.137	9.5467	2.5496	35.47	0.38	-6.53104	-6.03	0.00035	0.0009	yes
	T22C1.9		27.261	8.6707	1.6922	19.04	0.34	-5.79727	-5.1	0.0007	0.0018	yes
	T28H11.7		27.092	8.5009	2.6266	21.48	0.45	-5.56923	-5.3	0.00015	0.0004	yes
	K04G2.10		26.962	8.371	-0.4372	1.589	12	2.9119	6.228	0.00005	0.0001	yes
	T24D5.6		31.669	13.0784	-1.2758	3.406	21.4	2.64955	3.479	0.0002	0.0006	yes
	W06D11.3		26.274	7.6829	-1.4629	3.163	42.6	3.75117	8.961	0.00005	0.0001	yes
	F14H3.3		26.788	8.1968	-0.8978	2.395	20.8	3.1156	7.922	0.00005	0.0001	yes
	R09A8.9		29.397	10.8058	-1.1607	23.99	153	2.67018	2.623	0.00145	0.0035	yes
	C14C10.1	19.1111 Ref in CeHR	25.987	6.8755	-2.8454	53.43	0.79	-6.0817	-9.74	5.00E-05	0.0001	yes
	ZK938.1		25.211	6.0994	-1.9718	57.85	0.94	-5.94209	-10.9	0.00005	0.0001	yes
	K05F1.9		26.108	6.9971	-2.5496	35.47	0.38	-6.53104	-6.03	0.00035	0.0009	yes
	T22C1.0		26.09	6.9785	-1.6922	19.04	0.34	-5.79727	-5.1	0.0007	0.0018	yes
	T28H11.7		24.985	5.8743	-2.6266	21.48	0.45	-5.56923	-5.3	0.00015	0.0004	yes
	K04G2.10		27.919	8.8082	0.4372	1.589	12	2.9119	6.228	0.00005	0.0001	yes
	T24D5.6		33.465	14.3542	1.2758	3.406	21.4	2.64955	3.479	0.0002	0.0006	yes
	W06D11.3		28.257	9.1458	1.4629	3.163	42.6	3.75117	8.961	0.00005	0.0001	yes
	F14H3.3		28.206	9.0946	0.8978	2.395	20.8	3.1156	7.922	0.00005	0.0001	yes
	R09A8.9		31.078	11.9665	1.1607	23.99	153	2.67018	2.623	0.00145	0.0035	yes
						**CeHR**	**Rev**	**log2 (fold change)**	**test_stat**	**p-value**	**q-value**	**significant**
CeHR Vs Rev	R06F6.7	19.1111 Ref in CeHR	26.801	7.6894	-6.3797	10.69	0.73	-3.8752	4.609	4.1E-06	2E-05	yes
	F07B7.8		35.269	16.1582	0.4853	15.64	0.91	-4.10152	6.033	1.6E-09	1E-08	yes
	K04G11.1		35.119	16.0075	4.4802	1.292	0.14	-3.22564	2.263	0.02365	0.0457	yes
	F19F10.1		26.422	7.3105	-6.1145	19.65	2.36	-3.05698	7.443	9.8E-14	1E-12	yes
	F07D3.3		28.599	9.488	1.4454	12.49	1.62	-2.94789	6.395	1.6E-10	1E-09	yes
	T16G1.13		28.821	9.7096	-1.2957	0.263	7.76	4.88152	-2.52	0.01173	0.0245	yes
	R06F6.7	18.6704 Ref in Rev	29.703	11.0323	6.3797	10.69	0.73	-3.8752	4.609	4.1E-06	2E-05	yes
	F07B7.8		32.74	14.0691	-0.4853	15.64	0.91	-4.10152	6.033	1.6E-09	1E-08	yes
	K04G11.1		34.343	15.6729	-4.4802	1.292	0.14	-3.22564	2.263	0.02365	0.0457	yes
	F19F10.1		30.198	11.5273	6.1145	19.65	2.36	-3.05698	7.443	9.8E-14	1E-12	yes
	F07D3.3		32.095	13.425	-1.4454	12.49	1.62	-2.94789	6.395	1.6E-10	1E-09	yes
	K04G2.10		26.713	8.0426	-0.7656	1.271	63.7	5.64677	-10.1	0	0	yes
	T24D5.6		28.821	10.151	-4.2032	3.406	21.4	2.64955	3.479	0.0002	0.0006	yes
	W06D11.3		26.18	7.5094	-1.6364	3.163	42.6	3.75117	8.961	0.00005	0.0001	yes
	F14H3.3		26.616	7.9455	-1.1491	2.395	20.8	3.1156	7.922	0.00005	0.0001	yes
	T16G1.13		29.676	11.0053	1.2957	0.263	7.76	4.88152	-2.52	0.01173	0.0245	yes

Table S3: Oligos for qRT-PCR

C14C10.1
For: 5′- ACC ACA ATC ACC ATC TTC CA -3′
Rev: 5′- CCA TTC CGC ACA AGA CAT AG-3′

ZK938.1
For: 5′- AAT TCG ACA ACA ATG CAG GA-3′
Rev: 5′- ACT TCT CTC ATG GCT GGC TT-3′

K05F1.9
For: 5′- AGC TTG GCG ATG AAG TGA C-3′
Rev: 5′- TGA ATG CAT ACC GAT CCT TG-3′

T22C1.9
For: 5′- AAT TAG GTG CAT TGG GTG AAG-3′
Rev: 5′- CAC CAT GAA CAT CTT CCT TCT T-3′

T28H11.7
For: 5′- CAA GGA CCT TCA CAA GAG CA-3′
Rev: 5′- AAT TCT CAA GGG TGC CAG AG-3′

K04G2.10
For: 5′- GGA CGA GAG GTA CCT GAC AAA-3′
Rev: 5′- CTT CAT CTT CCG ATT CTC ACA T-3′

W06D11.3
For: 5′- AAC GAG CAA CAG GAT GGA G-3′
Rev: 5′- CGA GCT AGA AGT GTC CAC CA-3′

F14H3.3
For: 5′- GAG CGC AAG TTG GAT ATT GA-3′
Rev: 5′- TCT TCC GTC TCC TTC CTA TCA-3′

T24D5.6
For: 5′- GAC GAC AAA GGA TCG GAT G-3′
Rev: 5′- CTG TCC CGG AAC TAT CCA TT-3′

R09A8.9
For: 5′- ATA TGG TGG AGG GAT CAG GA-3′
Rev: 5′- TAG AAA GTC GTC GTC GTC CA-3′

R06F6.7
For: 5′- ACA ATC GTG TGA AGC GAG AT-3′
Rev: 5′- TTG CGA CGA ATA TCA TAG GC-3′

F07B7.8
For: 5′- CAT TCC CAG ATG CAG TTG AC3′
Rev: 5′- GTT AGC GCA TGC TTC GTT T3′

K04G11.1
For: 5′- CCT TGT TAC ATG GGC TTT CA-3′
Rev: 5′- TGT GGC AAT GAA TCA AGA GTT-3′

F19F10.1
For: 5′- TCC GTC TTC TGA GCT TCC TT-3′
Rev: 5′- CTT GTG TGG CTT GCT CAT CT-3′

F07D3.3
For: 5′- TCA AAC TCT GGA ATG GGT CA-3′
Rev: 5′- CAG CCT GCA TAC ACT CAT CC-3′

T16G1.13
For: 5′- TGG CAC GTA TCA ACT CGT TT-3′
Rev: 5′- GGC CTG TTG TCA TCT GTG TC-3′

ACT-1
For: 5′- AGC CAT CCT TCT TGG GTA TG-3′
Rev: 5′- ATT CCT GGG TAC ATG GTG GT-3′

Table S4. Gene expression comparison with a previous axenic medium study

(a) Genes downregulated in axenic medium versus OP50 NGM (red downregulated; green upregulated gene expression; FPKM > 1 if bolded)

Transcript Name	Gene Name	Reported (Szewczyk et al., 2006)	RNA-seq (N2)	RNA-seq (AB1)
F59A1.10	dgat-2	2.12±0.16	1.6763	1.34785
C18C4.5	C18C4.5	2.09±0.17	1.23525	0.338265
T21B10.3	T21B10.3	2.06±0.07	1.21561	1.07386
F42H10.7	ess-2	2.23±0.16	0.982566	0.33833
K05C4.5	K05C4.5	2.31±0.22	1.08071	1.13907
ZK856.9	zhit-3	2.23±0.06	0.315686	0.887203
ZC395.8	ztf-8	2.58±0.15	0.639296	0.696436
T10F2.3	ulp-1	2.82±0.23	1.28647	0.771096
ZK593.8	fic-1	2.96±0.29	0.915745	0.0010156
F22B7.5	dnj-10	2.36±0.15	0.302129	0.281979
K12D12.1	top-2	2.07±0.12	1.54555	1.60623
T07A9.6	daf-18	3.21±0.32	1.61265	0.586463
Y39A1A.12	orc-1	2.70±0.15	**2.0381**	0.694941
F49E8.2	oef-1	2.27±0.11	1.79849	0.96101
ZC317.7	ZC317.7	2.39±0.16	0.367506	0.540345
T02B5.1	cest-1	2.78±0.17	1.67789	**2.24475**
ZK1320.9	ZK1320.9	2.44±0.22	0.231765	0.39998
F25D1.1	ppm-1	3.90±0.28	0.708939	0.52062
F56A6.1	sago-2	3.88±0.35	2.45443	1.43725
C55B7.1	glh-2	2.46±0.18	1.84883	1.80556
C10H11.1	viln-1	2.41±0.20	0.14354	0.179035
K07D8.1	mup-4	2.74±0.15	1.1408	1.43152
T22B7.7	T22B7.7	4.90±0.20	1.02483	**2.65249**
F59D8.C	vit-3	3.82±0.38	Low reads	Low reads
C47B2.6	gale-1	3.01±0.24	0.275766	1.08986
F02A9.4	F02A9.4	3.63±0.32	1.0187	1.01102

Table S4 Cont'd

(b) Genes upregulated in axenic medium versus OP50 NGM (red downregulated; green upregulated gene expression; FPKM > 1 if bolded)

Transcript Name	Gene Name	Reported (Szewczyk et al., 2006)	RNA-seq (N2)	RNA-seq (AB1)
R13H8.1a	daf-16	2.11±0.11	0.361921	0.22918
T20D3.6	T20D3.6	2.33±0.19	0.621571	0.299947
F31E3.6	F31E3.6	2.27±0.16	1.98507	0.189954
F35A5.2	F35A5.2	3.95±0.29	**2.55678**	4.4825
C18A11.2	C18A11.2	3.58±0.10	**2.81051**	**3.66397**
C39D10.2	C39D10.2	2.46±0.17	1.30746	**3.62688**
M03E7.4	M03E7.4	2.31±0.19	1.6438	2.70823
T08G5.10	mtl-2	3.92±0.36	0.243858	**6.35372**
T10B10.6	phat-6	3.80±0.21	**2.20061**	5.70724
F32H5.3	F32H5.3	3.15±0.09	**3.3075**	**2.74311**
C25E10.8	C25E10.8	2.15±0.14	1.09099	1.716
K11G9.6	mtl-1	2.85±0.27	1.52386	**6.75533**
F18E9.2	nlp-7	2.14±0.18	0.930323	**2.02463**
F36D3.9	cpr-2	7.64±0.18	10.8343	**10.2444**
F54E7.2	rps-12	2.37±0.20	1.25192	0.694212
F37C12.9	rps-14	2.28±0.16	0.976764	0.221806
F37C12.11	rps-21	2.60±0.20	1.49071	0.675332
F39B2.6	rps-26	2.10±0.15	0.912086	0.276755
B0412.4	rps-29	2.47±0.16	1.1284	0.760152
Y48B6A.2	rpl-43	2.49±0.12	0.284669	1.0072
F28F8.3	lsm-5	2.53±0.13	0.929627	0.287242
Y46G5.36	Not found	2.84±0.11	Not found	Not found

Table S5. Differentially expressed long (purple), thin (pink), or long and thin (orange) phenotype genes under CeHR conditions in N2 and AB1 strains

N2		AB1	
**Upregulated in CeHR versus OP50 NGM**	**Downregulated in CeHR versus OP50 NGM**	**Upregulated in CeHR versus OP50 NGM**	**Downregulated in CeHR versus OP50 NGM**
adt-2	egg-6	cki-1	acs-1
C15A11.2	F56F11.4		his-67
his-3	frh-1		snrp-200
jph-1	his-62		spg-7
lon-8	his-67		Y105E8A.25
	rpb-8		
	vps-28		
	W05F2.3		
	Y95D11A.3		
	cyb-2.1		
	cyb-2.2		
	K08F4.3		
	kca-1		
	Y54G9A.5		

Supplementary Dataset Legends

Supplementary Dataset 1. Genes with genotype, environment, or genotype-environment interaction specific expressions

Supplementary Dataset 2. Commonly expressed ncRNA molecules in all three experiments
